# Machine learning-based tumor associated macrophages polarity signature predicts prognosis and treatment response in hepatocellular carcinoma

**DOI:** 10.3389/fimmu.2025.1663519

**Published:** 2025-11-05

**Authors:** Fangzhou Wang, Quan Zhang, Shichun Lu, Yamin Zheng

**Affiliations:** ^1^ Department of General Surgery, Xuanwu Hospital, Capital Medical University, Beijing, China; ^2^ Department of Oncology, Xuancheng City Central Hospital, Xuancheng, Anhui, China; ^3^ Medical School of Chinese People’s Liberation Army (PLA), Faculty of Hepato-Pancreato-Biliary Surgery, Chinese PLA General Hospital, Institute of Hepatobiliary Surgery of Chinese PLA, Key Laboratory of Digital Hepatobiliary Surgery, PLA, Beijing, China

**Keywords:** machine learning, hepatocellular carcinoma, macrophages polarity, prognosis, single-cell RNA-seq

## Abstract

**Background:**

Tumor-associated macrophages (TAMs) shape the tumor microenvironment and drive hepatocellular carcinoma (HCC) progression. However, the prognostic significance of TAM polarity-related genes, particularly based on the CXCL9:SPP1 signature, remains unclear.

**Methods:**

We identified 372 TAM polarity-related genes in the TCGA-LIHC dataset. Prognostic candidates were selected using univariate Cox regression, bootstrap resampling, and the Boruta algorithm. Seven machine learning models were compared, and XGBoost was selected to construct a TAM polarity-related signature (TPS) consisting of 17 genes. TPS was validated in two external cohorts. Associations with clinical features, biological pathways, immune status, and drug sensitivity were explored. scRNA-seq and qRT-PCR were performed to investigate cellular expression and functional relevance.

**Results:**

TPS markedly different patients into high- and low-risk groups with significantly different survival outcomes (TCGA 1-, 3-, 5-year AUCs: 0.91, 0.89, 0.88). High-risk patients showed enrichment in glycan metabolism, DNA repair, and oncogenic pathways, whereas low-risk patients displayed elevated lipid and amino acid metabolism. Immune profiling revealed greater infiltration of immunosuppressive cells and higher expression of immune checkpoints in high-risk patients. Drug sensitivity analysis identified potential therapeutic targets and candidate compounds, including CDK1, PLK1, and statins. scRNA-seq analysis highlighted disrupted macrophage-immune interactions and identified SPP1 as a key signaling mediator. Silencing of TTC1 and G6PD suppressed HCC cell proliferation.

**Conclusion:**

We developed and validated a robust TAM polarity-related signature that effectively stratifies HCC patients by prognosis. TPS provides insights into tumor immunity, metabolism, and drug response, and may serve as a valuable tool for precision medicine in HCC.

## Introduction

Hepatocellular carcinoma (HCC) is the most common type of primary liver cancer and the second leading cause of cancer-related death globally. Due to the absence of specific symptoms, nearly 50% of patients are diagnosed with advanced-stage HCC. Current systemic therapies, including immune checkpoint inhibitors, antiangiogenic targeted drugs, transarterial chemoembolization and other treatments, yield an objective response rate of only 45-55% (1, 2). Consequently, there is an urgent need for new therapeutic targets and strategies for patients with advanced HCC.

The advent of high-throughput sequencing has enabled early identification of HCC patients with a poor prognosis and a suboptimal treatment response, facilitating personalized therapies to improve outcomes. The tumor microenvironment (TME), comprising cancer cells, immune cells, and diverse stromal elements, plays a crucial role in cancer progression. Interactions within this ecosystem significantly influence cancer development and treatment responses (3–5). Deciphering the TME is therefore critical to understanding cancer biology.

Several studies have focused on tumor-associated macrophages (TAMs) and the TME, which are predominantly of the M2 phenotype within tumors and are associated with immunosuppression and negative immune regulation. For instance, Yang highlighted TAM-related mechanisms in immunotherapy (6). Han and Zhou identified pathways driving M2 macrophage polarization in HCC (7, 8). Recent single-cell RNA sequencing studies revealed that TAM polarity, defined by CXCL9:SPP1 expression, has significant value in head and neck squamous cell carcinoma. This CXCL9:SPP1 polarity correlated with increased infiltration of immune cells, and is associated with increased T cell abundance (9).

Numerous studies have derived prognostic signatures in HCC based on the TME. For example, NK cell–centric models have stratified patients by survival and predicted immunotherapy sensitivity by capturing immune exhaustion versus active anti-tumor states (10). TME-wide risk scores have delineated immune subtypes and suggested patients with low TMErisk may benefit from immune checkpoint inhibitors whereas those with high TMErisk appear more suited to multi-TKIs (11). Other frameworks emphasized stromal programs such as cancer-associated fibroblasts to predict immunotherapy response and drug sensitivity and Hallmark-guided immune classifiers and immune checkpoint related gene signatures provided robust prognostic tools (12, 13). Collectively, these models underscore the value of immune contexture in HCC and may enhance our understanding of the functional role of TAM within the TME.

HCC progression often coincides with an immunosuppressive TME, driven by chronic inflammation, fibrosis and cirrhosis (14). Therefore, evaluating TAM polarity in HCC patients is essential for precise treatment planning. CXCL9:SPP1 TAM polarity has been observed in non-small cell lung cancer and colorectal cancer, its transcriptomic signature has not been thoroughly investigated in HCC. This study aims to classify TAM polarity at the transcriptomic level and to develop a predictive model for HCC patients. Machine learning techniques are increasingly applied in bioinformatics and oncology research due to their superior predictive capabilities in managing high-dimensional and heterogeneous clinical data. However, recent studies have highlighted variations in the performance and stability among different algorithms. As a result, it is crucial to select the most suitable algorithm based on established benchmarks for clinical decision-making.

Our study established a robust TAM polarity signature using seven machine-learning algorithms. This signature demonstrated superior predictive power compared to other signatures and clinical variables across independent cohorts. Additionally, we identified potential therapeutic targets and agents through in silico analysis and explored immune interactions and cell cross-talk using sc-RNA sequencing. We validated the expression of TTC1 and G6PD at both the transcriptomic and proteomic levels and investigated their effects on cell proliferation.

## Materials and methods

### Data collection and processing

The Cancer Genome Atlas (TCGA) database was used for the initial analysis. Specifically, mRNA expression data and clinical information from the TCGA-LIHC cohort (n = 365) were obtained from UCSC XENA in March 2024. Additionally, external datasets, including GSE14520 (n = 221), LIRI_JP (n = 130), were obtained from the Gene Expression Omnibus and International Cancer Genome Consortium (ICGC). Only HCC samples with available survival data were included across all datasets. For somatic mutation analysis, the maftools package (v2.22.0) was used. RNA sequencing data were processed using log2(transcripts per million + 1) transformation to represent mRNA expression levels. For raw expression data obtained from microarrays, background adjustment and normalization were performed using the Robust Multi-Array average algorithm from the ‘affy’ R package (v1.82.0). To address batch effects across cohorts, the ‘ComBat’ function from the ‘sva’ R package (v3.56.0) was used to correct batch effects and integrate the data into a Meta-cohort (**Supplementary Tables S1**, **S2**). Both overall survival and biochemical recurrence survival were considered as primary endpoints. Additionally, drug sensitivity data were retrieved from Cancer Cell Line Encyclopedia project and the secondary PRISM Repurposing dataset, encompassing 1,448 compounds tested against 489 cancer cell lines. Raw single-cell RNA sequencing FASTQ data was collected from samples at PLA general hospital, Beijing, China (EGAC00001001616 and GSE149614).

### Construction of TAM polarity-related signature by machine learning benchmark

Based on previous study (9), 1812 TAM polarity related genes were selected, and genes do not present in the HCC bulk RNA datasets were excluded. Subsequently, univariate Cox regression analysis was performed using the “Survival” package (v3.7) in the TCGA-LIHC cohort to identify genes associated with prognosis (P<0.01). A bootstrap approach, sampling 80% of cases 1,000 times, was employed to assess the robustness of the selected genes. Furthermore, the Boruta algorithm with ntree = 1,000 and maxRuns = 1,000, was used to further identify genes most strongly associated with prognosis by comparing the importance of selected features and random ones.

To ensure the accuracy and the robustness of risk signature, we employed 7 benchmark machine learning algorithms, including Lasso, Elastic network, Ridge, Partial least squares regression for Cox (plsRcox), CoxBoost, eXtreme Gradient Boosting survival (XGBoost), and Supervised principal components (SuperPC), using nested cross-validation (CV) in TCGA-LIHC cohort. The inner fold and outer fold hyper-parameters of these algorithms were set to 5-fold and 10-fold, respectively, to evaluate the performance of the best-tuned algorithm (**Supplementary Tables S3**-**S5**). Harrell`s concordance index (C-index), integrated Brier score (IBS), and 1-, 3-, 5- and 10-year area under the ROC curve (AUC) values from 10 testing folds were used to comprehensively evaluate the performance of 7 models. The model with the highest average metrics was selected as the optimal model, and the function “xgb.importance” in XGBoost (v 1.7.6) was used to evaluate the contribution of each feature to the model predictions.

### Comparison of TAM polarity-related signature with published signatures

To ensure the robustness of risk signature, we compared our signature with 4 HCC signatures from previous studies, including Lu et al. (single-cell RNA signature), Zhang et al. (hypoxia-related signature), Genhao Zhang (regulatory T-cell related signature) and Zhao et al. (immune checkpoint related signature) (**Supplementary Table S6**) (15–18). We applied each published model`s gene coefficients to datasets to calculate a risk score for each sample. The C-index and AUC values of each signature were computed and compared.

### Differential expression analysis

Differential analysis between high-risk and low-risk groups was performed using the “Limma (v3.54.2)” R package. Genes with an adjusted P value < 0.05 and absolute log2 (Fold-change) > 1 were considered differentially expressed.

### Gene set enrichment analysis and immune cell infiltration

To investigate the relationship between the risk signature and biological pathways, we utilized single-sample gene set enrichment analysis (ssGSEA) to calculate activity scores for both the Hallmark and Kyoto Encyclopedia of Genes and Genomes (KEGG) gene sets (h.all. v7.4. symbols and c2.cp.kegg. v7.4. symbols, **Supplementary Tables S9**-**S11**). These scores were computed on the Meta-cohort using the ‘GSVA’ R package. Additionally, to examine the influence of TPS on the TME, the CIBERSORT algorithm was used to estimate the proportions of 22 tumor-infiltrating immune cell types in the Meta-cohort.

### In silico discovery of potential targets for high-risk HCC patients

Because many proteins lack suitable binding sites or do not exhibit sufficient affinity for small molecules or antibodies, we initially gathered 2,249 druggable targets from a prior study (**Supplementary Table S13**). We the performed a Spearman’s rank-order correlation analysis to identify targets positively correlated with the risk score, using threshold of correlation coefficient > 0.3 and false discovery rate (FDR) < 0.05. Targets with CERES scores greater than -1 in multiple HCC cell line were excluded.

To identify potential therapeutic agents, we first used the Connectivity MAP (CMap), which compares a differential gene signature to perturbation signatures in its database and generates a similarity score. Drugs with scores below -95 were considered potential candidates for reversing the high-risk gene expression signature. Additionally, using the “pRRophetic” R package, we trained a Ridge regression model with 10-fold cross-validation to predict drug sensitivity, leveraging transcriptomic data and corresponding drug response data from the PRISM dataset. This model was then applied to predict the AUC of each drug for each HCC sample based on its transcriptomic profile. Also, we validated it using a dedicated cohort of patients with HCC who underwent anti-PD-1/PD-L1-based immunotherapy (GES202069).

### Single-cell sequence data analysis

The scRNA-seq data were initially filtered using the Seurat R package (v4.3.0). We excluded cells with fewer than 300 or more than 6,000 expressed genes, or with over 10% of unique molecular identifiers mapping to mitochondrial genes. Only genes expressed in at least five cells were retained. The data were normalized, and the “FindVariableFeatures” function was used to identify the top 2,000 highly variable genes. Dimensionality reduction was performed via PCA on these variable genes, and the first principal components were selected for subsequent analyses. Batch effects across samples were corrected using the Harmony R package. Finally, cells were clustered using the “FindClusters” function with a resolution of 0.5. Cell types were identified based on annotations from the CellMarker database and relevant literature.

### Cell crosstalk analysis

To investigate the communication patterns between cells, we used the CellChat R package. Following the recommended workflow, we first generated a CellChat object from a normalized count matrix. We then applied the “identifyOverExpressedGenes” and “identifyOverExpressedInteractions” functions with default parameters for preprocessing. Potential ligand-receptor interactions among all cell types, with a particular focus on interactions between immune cells and tumor cells, were inferred using the functions “computeCommunProb”, “computeCommunProbPathway”, and “aggregateNet” functions with default settings.

### Validation of TAM polarity-related genes expression level

A total of 12 paired specimens of HCC and adjacent non-tumor tissues were obtained from the First Medical Centre of the Chinese PLA General Hospital (**Supplementary Table S18**). After excision, all specimens were promptly preserved in liquid nitrogen at -196°C. Total RNA was extracted using TRIzol reagent (Invitrogen, California, USA), and complementary DNA was synthesized using the ReverTra Ace qPCR RT Kit (Toyobo, Japan). Quantitative PCR detection of the prognostic genes was performed with SYBR^®^ Green Realtime PCR Master Mix (Toyobo, Japan) on the ABI Step One Plus Real-Time PCR system (Applied Biosystems), using β-Actin as an endogenous control. Each sample was tested in triplicate. The primer sequences were as follows: TTC1-F (5′-GAGCGGACAAGGTTGAGAACA-3′),TTC1-R (5′-CTCCTCCTTTAGTCTAGTGCTCT-3′), G6PD-F (5′-CGAGGCCGTCACCAAGAAC-3′), G6PD-R (5′- GCATGGGTCAGAAGGATTCCTATGT-3′), ACTB-F (5′-GAGCGGACAAGGTTGAGAACA-3′) and ACTB-R (5′- CGGTGAGGATCTTCATGAGGTAGT-3′). Differences in gene expression between HCC and corresponding adjacent non-tumor tissues were analyzed using the 2^−ΔΔCt^ method. In addition, we examined the protein expression levels of TTC1 and G6PD in HCC using data from He and CPTAC dataset (Clinical Proteomic Tumor Analysis Consortium) (19). Finally, we performed Western blot analysis on HCC samples to validate TTC1 and G6PD protein levels. Briefly, tissues were rinsed with ice-cold phosphate-buffered saline (PBS) and lysed in a buffer supplemented with protease and phosphatase inhibitors. Equal amounts of protein lysate were separated on 10% polyacrylamide gels and transferred onto polyvinylidene difluoride membranes. Membranes were blocked in 5% non-fat milk for 1 hour at room temperature, then incubated overnight at 4 °C with primary antibodies. After three washes in TBST buffer, membranes were incubated with rabbit secondary antibody for 2 hours at ambient temperature. Protein bands were visualized using a chemiluminescence substrate and captured with a Tanon 5200 automatic imaging system (Shanghai, China). The primary antibodies used were anti-G6PD (ABclonal, Hubei, China; Cat#ab133525), anti-TTC1(Thermo-Abnova, Shanghai, China; Cat# abs118292), and anti-GAPDH (Cell Signaling Technology, Beverly, MA, USA; 1:2000, Cat# CST5174S).

### Cell culture and transfection

HepG2 and Hep3B cancer cell lines were maintained in DMEM medium supplemented with 10% fetal bovine serum and 1% penicillin-streptomycin solution and incubated under standard conditions at 37°C in a humidified atmosphere containing 5% CO_2_. For gene silencing, small interfering RNAs (siRNAs) were obtained from Genepharma and introduced into cells using Lipofectamine RNAiMAX (Thermo Fisher Scientific) according to the manufacturer’s protocol. Cells were harvested 48–72 hours post-transfection for downstream assays. The siRNA sequences were: siTTC1 (5′-UAUAGAUUUAUAGUCUUCCAG-3′) and siG6PD (5′-UUCUUGGUCAUCAUCUUGGUG-3′). Knockdown efficiency was confirmed by Western blot analysis as described above.

### Cell viability analysis and wound healing assay

Following transfection, HepG2 and Hep3B cells were seeded into 96-well plates at a density of 3 × 10³ cells per well. Cellular viability was assessed using the Cell Counting Kit-8 (Beyotime, Shanghai, China) according to the manufacturer’s guidelines. Absorbance at 450 nm, indicative of metabolic activity, was measured using a microplate reader (Thermo Fisher Scientific, California, United States) at time points of 0, 24, 48, 72, and 96 hours. The cell migration ability of HepG2 and Hep3B cells was evaluated using a wound healing assay. Briefly, cells were seeded in 24-well plates at a density of 5×10^5^ cells per well and cultured in DMEM supplemented with 10% FBS until a confluent monolayer formed. A uniform scratch was created using a sterile 10 μL pipette tip. Dislodged cells were removed by washing with PBS. Serum-free medium was then added to minimize cell proliferation. Images of the scratch were captured at 0 h and 24 h of incubation. The migration distance was quantified by measuring the change in scratch width using ImageJ software, and the percentage of wound closure was calculated to assess call migration capacity.

### Statistical analysis

Comparisons of continuous variables were conducted using the Kruskal-Wallis test or the Mann-Whitney U test, as appropriate. Categorical variables were expressed as counts and compared using the chi-square test or Fisher’s exact test. Survival curves were generated using the Kaplan-Meier method and compared by the log-rank test. Univariate and multivariate Cox regression analyses were used to identify independent risk factors for overall survival (OS). A p-value < 0.05 was considered statistically significant, and all statistical tests were two-tailed. Data analysis was performed using R version 4.1.0, SPSS 29.0, and GraphPad Prism 10.

## Results

### Overview of study design

**Figure 1:**
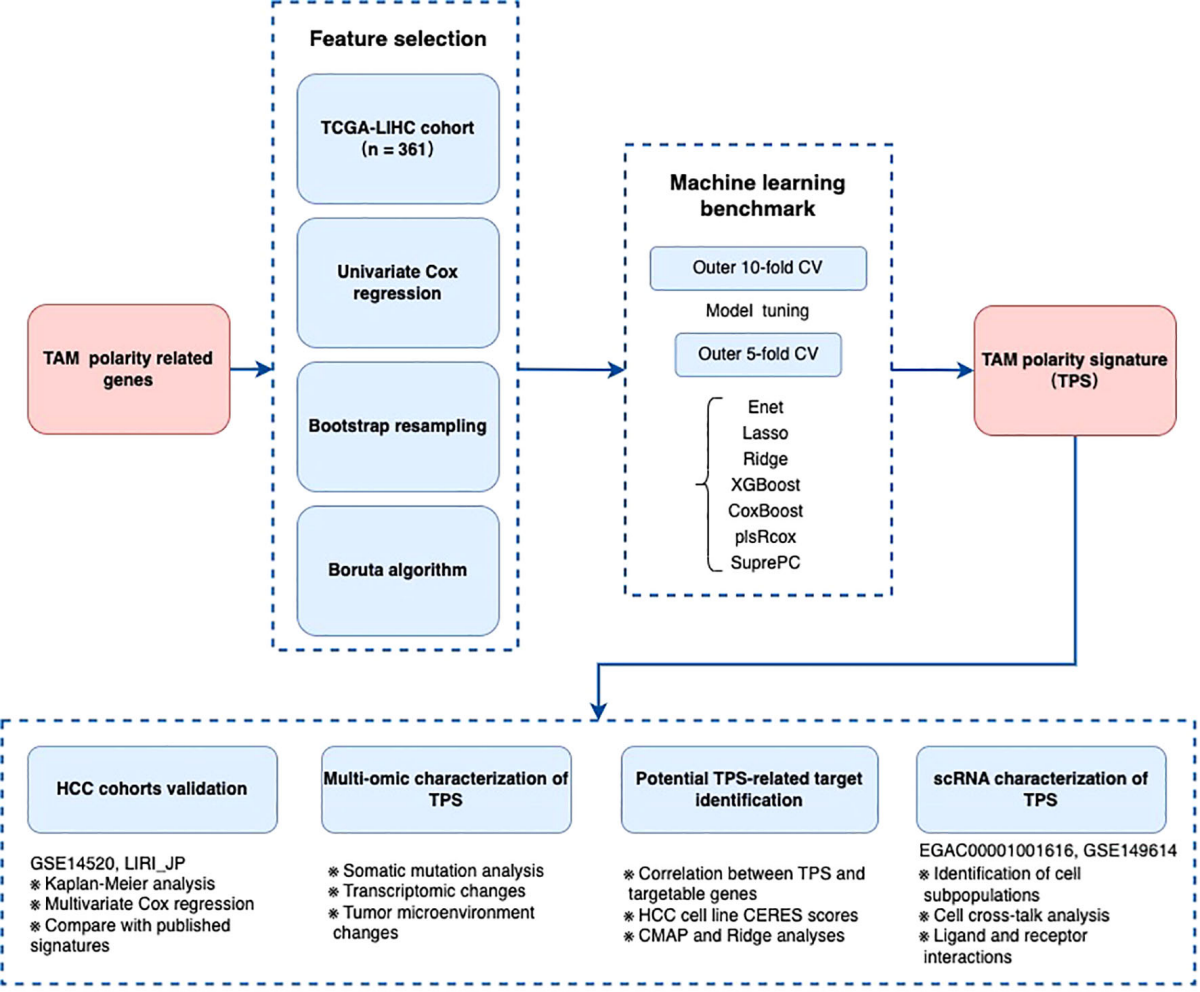
The flow chart of the establishment of TAM polarity signature in Hepatocellular carcinoma.

**Figure 1** presents an overview of study design, outlining the process from initial data collection to risk signature development and downstream analyses (**Supplementary Figure S1**).

### Identification of TAM polarity-associated signature features in TCGA-LIHC

**Figure 2:**
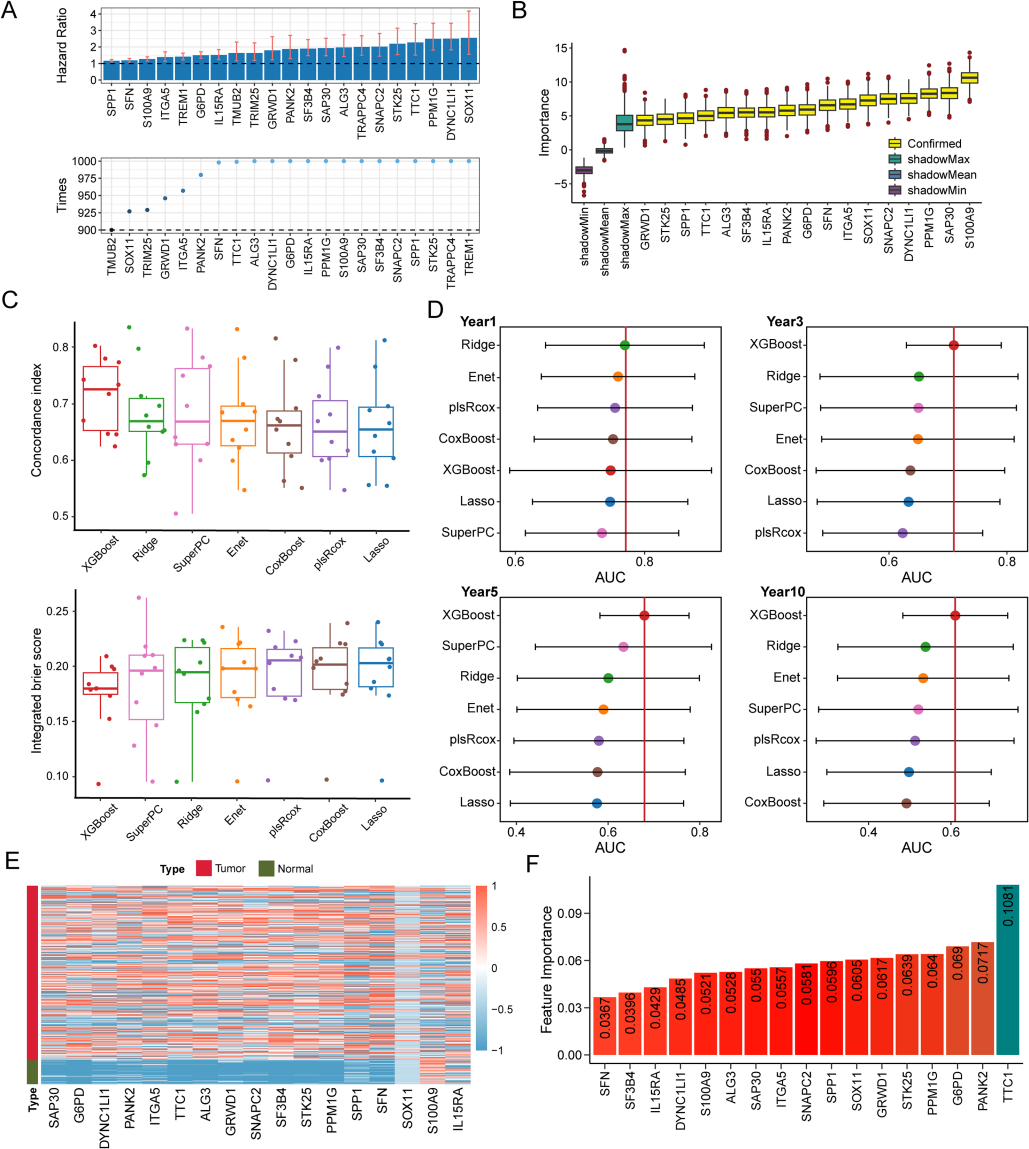
TAM polarity signature (TPS) was established utilizing machine learning benchmarks. **(A)** 17 TAM polarity related genes that associated with poor prognosis identified by the Boruta algorithm. Yellow denotes confirmed features and other color represent shadow attributes. **(B)** The boxplots compared the C-index values. **(C)** IBS of 7 survival related machine learning algorithms employed in validation. **(D)** Comparison of time dependent area under the receiver operating characteristic curve (AUC) at 1-, 3-, 5- and 10-year among 7 machine learning algorithms. The dots represent the average AUC values for each time point. **(E)** Heatmap indicates the expression of the signature gene in tumors and normal tissues. **(F)** A bar plot illustrating the feature importance of genes contributing to prostate cancer recurrence within the XGBoost model, based on data from the TCGA-LIHC cohort.

To identify TAM polarity-associated gene signatures, we began with 1,812 candidate genes from a previous study, of which 372 were present in the TCGA-LIHC dataset. Using univariate Cox regression on 365 cases in the TCGA-LIHC cohort, we identified a subset of genes significantly associated with patient prognosis. We further refined this list by applying a bootstrap selection approach in the validation datasets, followed by the Boruta algorithm to pinpoint the most prognostically important genes. As shown in **Figures 2A, B**, the Boruta analysis ranked 17 genes by importance, with TTC1, PANK2, G6PD, PPM1G, and STK25 emerging as the top five (**Figure 2**).

### Construction of TAM polarity-related signature

Using the 17 genes selected by Boruta, we evaluated seven prognostic modeling algorithms, Lasso, Ridge, Elastic Net, XGBoost, plsRcox, SuperPC, and CoxBoost, to identify which model provided the best predictive performance (**Supplementary Tables S5**). We implemented a nested cross-validation strategy, with an outer 10-fold cross-validation for model evaluation and an inner 5-fold cross-validation for hyperparameter tuning. As shown in **Figure 2C**, the XGBoost-based survival model achieved the highest performance across multiple metrics, including concordance index (C-index), integrated Brier score (IBS), and area under the curve (AUC). The TCGA-LIHC cohort was used as the training set for the XGBoost model (**Figure 2D**). As a result, we established a 17-gene prognostic risk signature, which included key contributors such as TTC1, PANK2, G6PD, PPM1G, and STK25 (**Figures 2E, F**).

### Evaluation of TAM polarity-related signature

**Figure 3:**
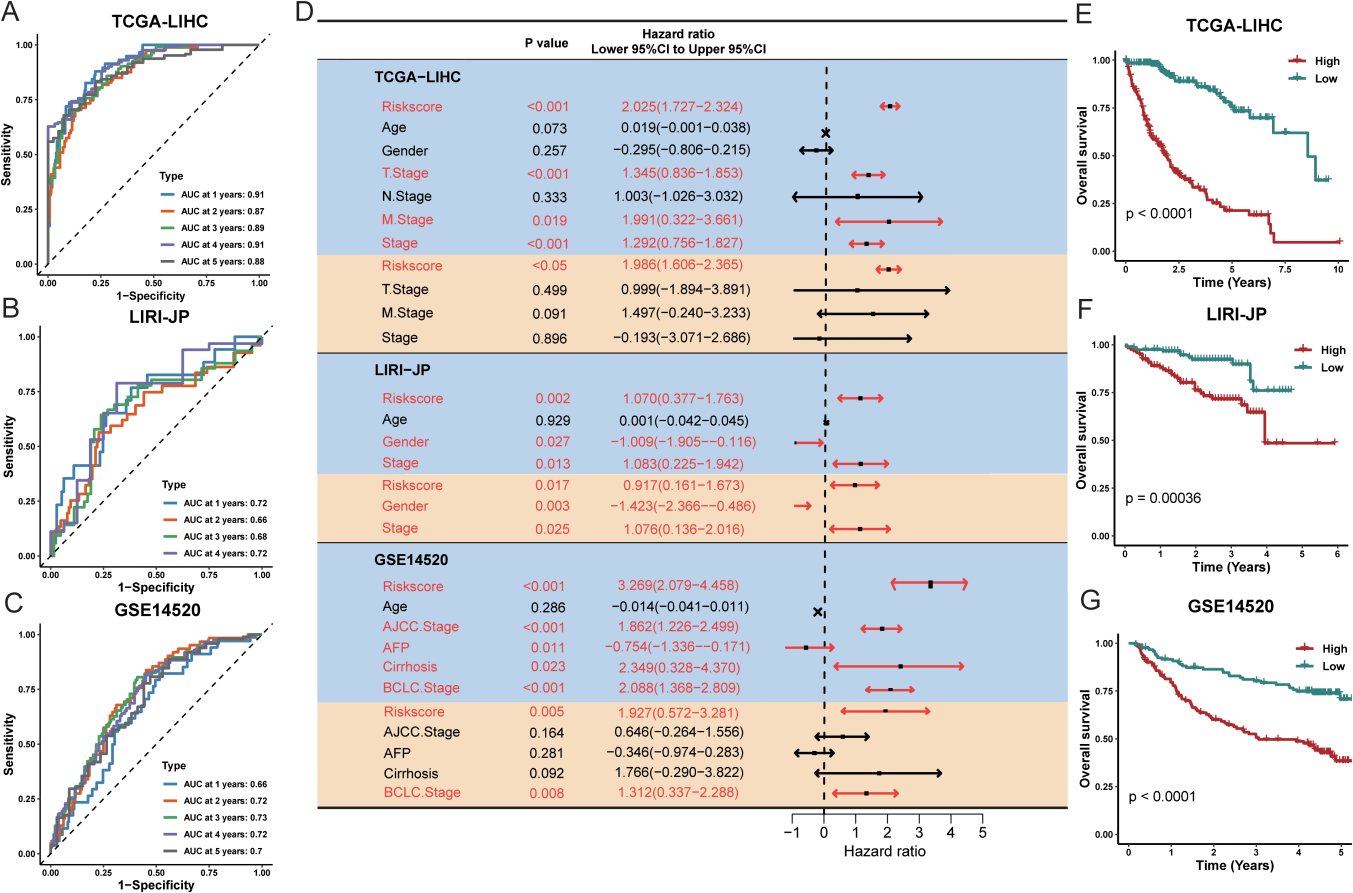
Evaluation of the TPS was conducted across multiple cohorts. The time-dependent area under the receiver operating characteristic curve (AUC) was assessed at 1, 3, and 5 years for the following datasets: **(A)** TCGA-LIHC, **(B)** LIRI-JP, and **(C)** GSE14520. **(D)** Forest plots illustrate the hazard ratio (HR), 95% confidence interval (CI), and corresponding P-values for both univariate (orange shading) and multivariate (blue shading) Cox regression analyses across five prostate cancer cohorts. **(E–G)** Kaplan–Meier plots display survival outcomes for TCGA-LIHC, LIRI-JP, and GSE14520, respectively. High- and low-risk groups were classified based on a universal cutoff of. P-values were obtained using the log-rank test. AFP refers to alpha-fetoprotein; TPS stands for TAM polarity signature.

We next assessed the prognostic significance of this risk signature in the TCGA-LIHC training cohort and two external validation cohorts (LIRI-JP and GSE14520). Performance was evaluated by the time-dependent AUC at 1, 3, and 5 years, as well as the concordance index (**Figures 3A–C**).

Univariate and multivariate Cox regression analyses in all three cohorts indicated that the risk signature was significantly associated with overall survival (**Figure 3D**), suggesting it is an independent prognostic factor for HCC. Specifically, in multivariate analysis for the TCGA-LIHC, LIRI-JP, and GSE14520 cohorts, the hazard ratios were 1.986 (95% CI: 1.606–2.365, P < 0.05), 0.917 (95% CI: 0.161–1.673, P = 0.017), and 1.927 (95% CI: 0.572–3.281, P = 0.005), respectively (**Figures 3E–G**). These findings indicate that the 17-gene signature effectively stratifies patients into high-risk and low-risk groups. Time-dependent ROC analysis and Kaplan Meier survival curves further demonstrated significant differences in survival between the high-risk and low-risk groups in all three cohorts. Overall, our TAM polarity-related risk signature showed strong and robust prognostic value for risk stratification in HCC patients.

### Comparison of TAM polarity-related signature to clinical variables and published signatures

**Figure 4:**
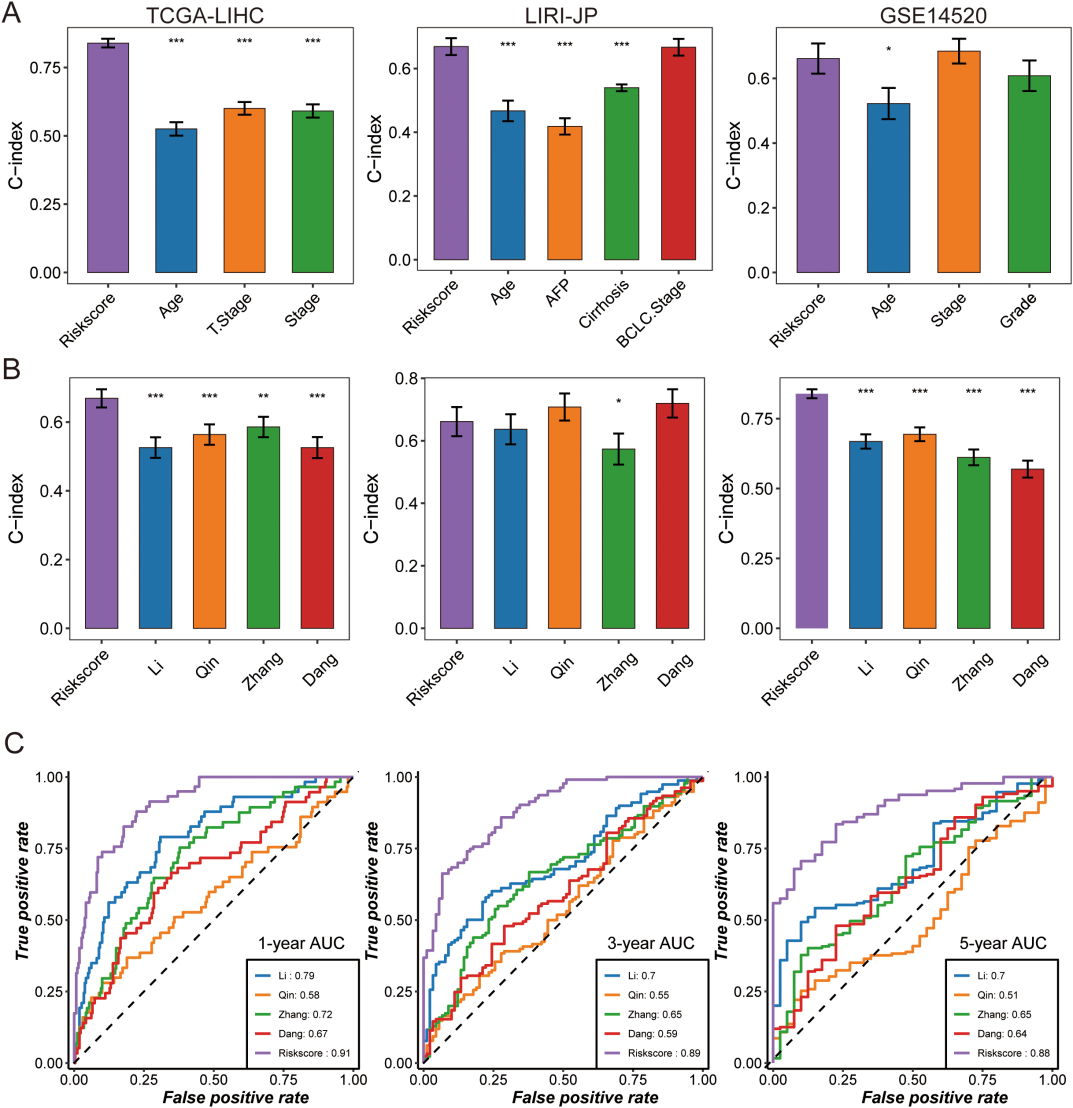
The predictive performance of TPS was compared with that of clinical features and other prognostic signatures. **(A)** The C-index was evaluated to compare TPS with clinical features in the following datasets: TCGA-LIHC, DKFZ-PRAD, and GSE14520. Data are presented as the mean ± 95% confidence interval. **(B)** Univariate Cox regression analysis of prognostic signatures was performed across three HCC cohorts, where dots represent log_2_ (hazard ratio), and the upper and lower bounds of the bars indicate log_2_ (95% confidence interval). The C-index was compared between TPS and other prognostic signatures across cohorts, with dots representing the mean C-index and bars indicating the 95% confidence interval. Time-dependent AUC was compared among prognostic signatures at **(C)** 1-year, 3-year, and 5-year intervals in the TCGA-LIHC dataset. Asterisks indicate statistical significance (* p < 0.05, ** p < 0.01, *** p < 0.001).

With the rapid development of next-generation sequencing technologies and the growing importance of genomics in clinical practice, robust prognostic biomarkers are increasingly needed. In this context, the risk score derived from our TAM polarity signature consistently demonstrated high predictive accuracy. In the TCGA-LIHC cohort, our signature (C-index 0.77) outperformed all traditional clinical variables as a survival predictor, and it showed similarly strong performance in the LIRI-JP cohort (C-index 0.68). In the GSE14520 cohort, the risk score (C-index 0.61) performed comparably to the best clinical indicator (tumor stage, C-index 0.63) (**Figure 4**).

We also compared our signature against other published HCC prognostic models by examining the C-index in the TCGA-LIHC, LIRI-JP, and GSE14520 datasets (**Figure 4**, **Supplementary Tables S8**). Our 17-gene signature achieved higher C-index values than the other models in all three cohorts. Consistently, ROC curves showed that the 1-year AUC of our risk score was 0.91, substantially higher than those of the comparator models, underscoring its superior and stable discriminative ability (**Figure 4**).

### Association of TAM polarity-related signature with clinical features and biological process

**Figure 5:**
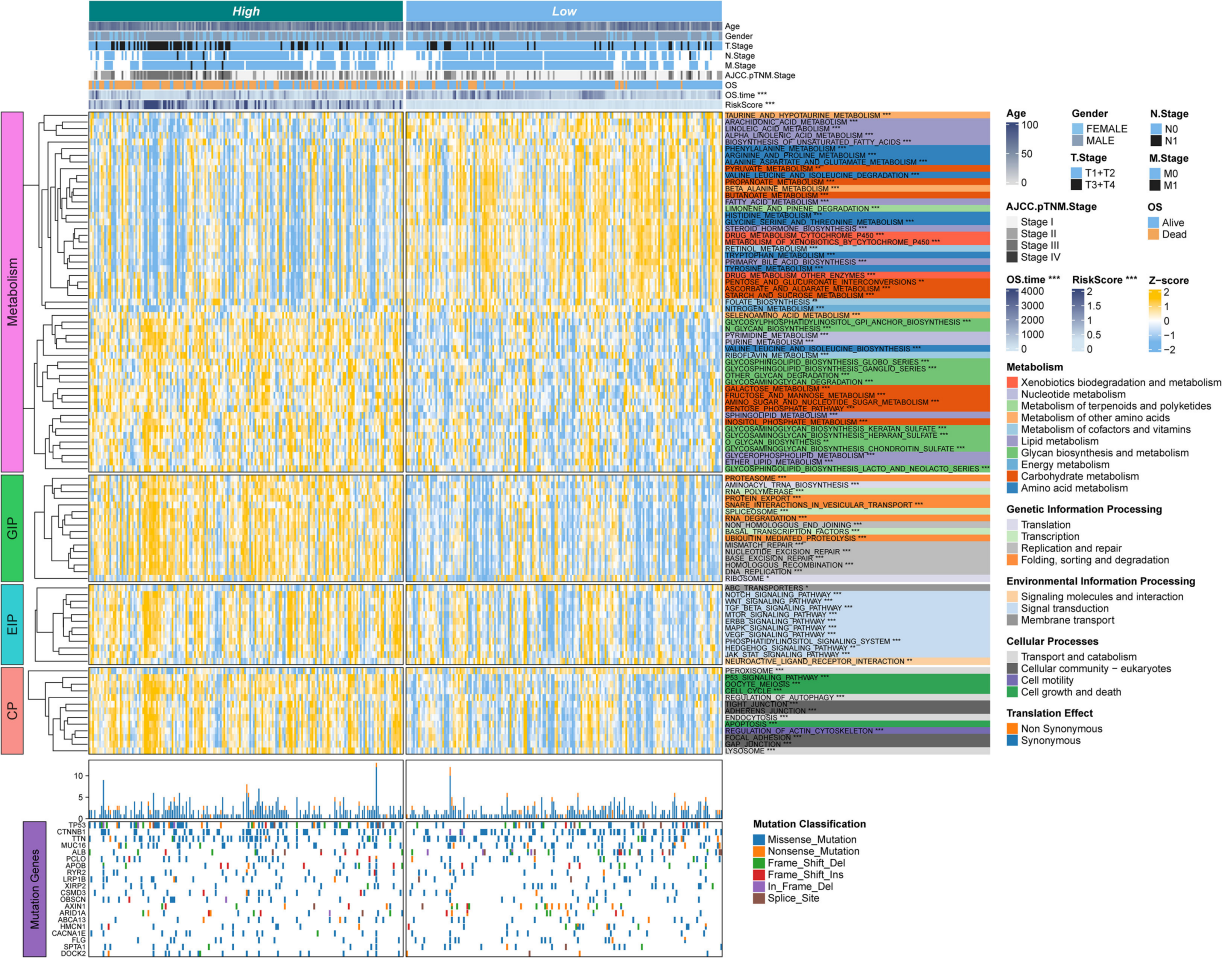
The associations between clinicopathologic and biological features and TPS were analyzed. The upper panel of the heatmap illustrates the distribution of clinical characteristics between RSS-high and TPS-low patients. The lower panel presents z-scores from ssGSEA. The color-coded right-side annotations indicate the relative enrichment of pathways in the corresponding groups, while the asterisk annotations denote statistical P-values. A multi-omic characterization was performed to compare TPS-high and TPS-low patients. An oncoprint of common somatic gene mutations is shown in the lower part, with the bar plot on the right indicating the proportion of somatic mutations in each group.

We next examined associations between the risk signature and clinical features, as well as its relationship to biological pathways. Across all cohorts (**Figure 5**), patients in the high-risk group tended to have more advanced disease, including higher tumor T stage and later overall stage (AJCC Stage III–IV), compared to those in the low-risk group (**Supplementary Figure S2**, **Supplementary Table S4**). To explore pathway differences, we performed single-sample Gene Set Enrichment Analysis (ssGSEA) on transcriptomic data from high- vs. low-risk patients. The high-risk group showed significant enrichment of glycan biosynthesis and carbohydrate metabolism pathways (e.g., glycosphingolipid biosynthesis, O-glycan biosynthesis, galactose metabolism; all P < 0.05) (**Figure 5**). In contrast, the low-risk group exhibited greater activity in lipid and amino acid metabolism pathways, such as arachidonic acid metabolism, linoleic acid metabolism, α-linolenic acid metabolism, and unsaturated fatty acid biosynthesis (all P < 0.05).

In addition, the high-risk group was significantly enriched in pathways related to genetic information processing and cell cycle regulation. These included multiple DNA replication and repair mechanisms, as well as apoptosis. Several oncogenic signaling pathways were also upregulated in high-risk tumors, notably Notch, WNT/β-catenin, TGF-β, and ERBB signaling. Meanwhile, the low-risk group showed higher enrichment of pathways involved in molecular transport and signaling, such as ABC transporters and neuroactive ligand–receptor interaction. Collectively, these findings suggest that high-risk HCC tumors are characterized by enhanced proliferative and metabolic activity, whereas low-risk tumors exhibit a different metabolic profile reflecting distinct metabolic reprogramming in HCC.

### Association of TAM polarity-related signature with the immune microenvironment

**Figure 6:**
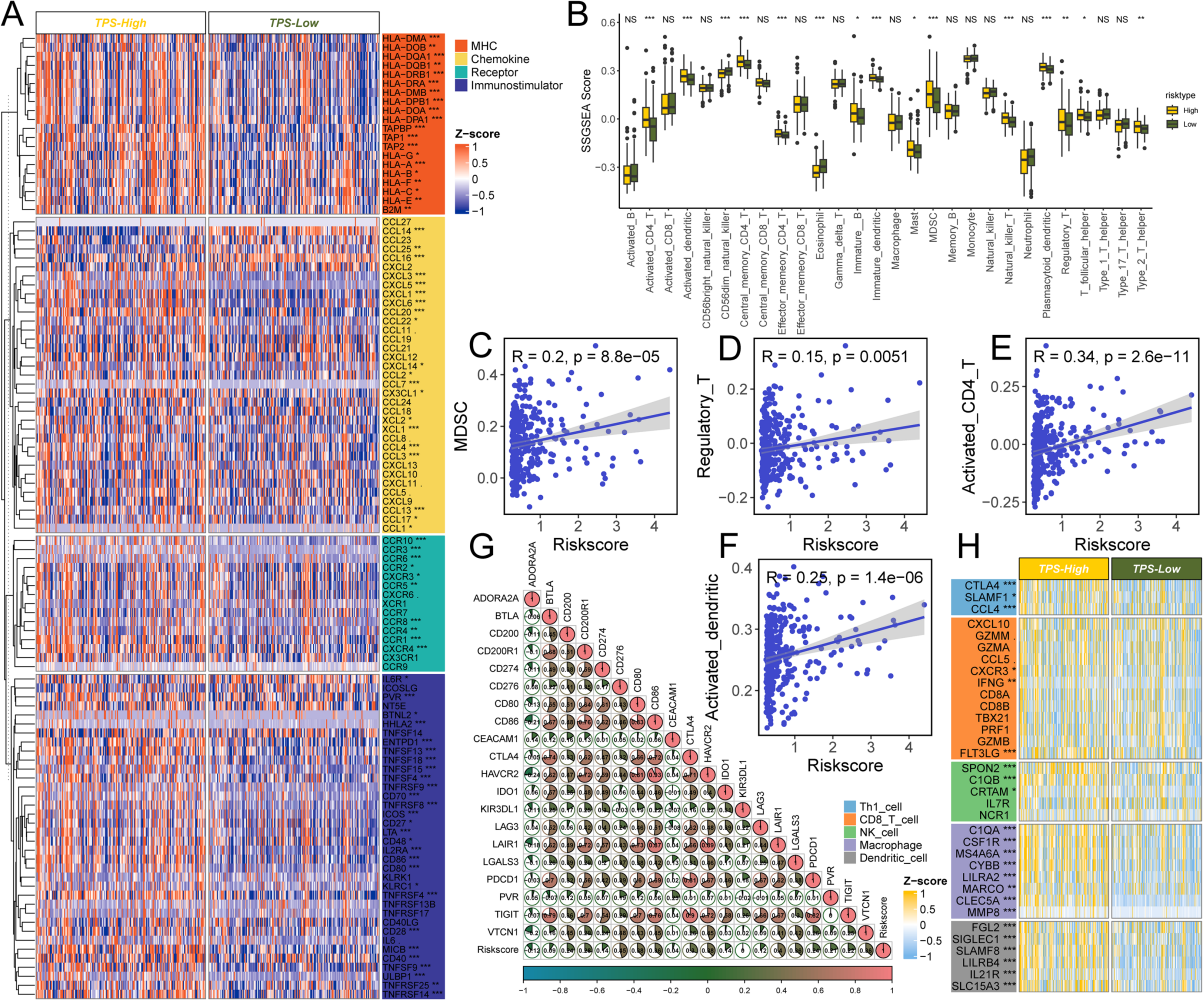
The effect of TPS on immunological status in HCC. **(A)** Differences in the expression of 122 immunomodulators (chemokines, receptors, MHC, and immunostimulators) between TPS-high and -low groups in HCC. **(B)** Differences in the various type of immune cells between TPS-high and low groups. **(C–F)** Correlation between TPS and the infiltration levels of five types of immune cells (MDSC, Regulatory T cell, Activated CD4^+^ T cell, and activated dendritic cells). **(G)** Correlation between TPS and 20 inhibitory immune checkpoints. The color and the values indicate the Spearman correlation coefficient. The asterisks indicated a statistically significant p-value calculated using Mann-Whitney U test (*P < 0.05; **P < 0.01; ***P < 0.001). **(H)** Differences in the effector genes of the above tumor-associated immune cells between TPS-high and -low groups.

Because TAMs are key regulators of tumor immunity, we investigated the immune landscape associated with the risk signature. Using CIBERSORT, we quantified immune cell infiltration in 365 HCC samples (**Supplementary Tables S8**, **S9**) and compared high- vs. low-risk groups. The high-risk group had elevated expression of many immunomodulatory molecules (**Figure 6A**). High-risk group showed increased expression of most MHC class I and II genes, suggesting an enhanced capacity for antigen presentation. They also exhibited upregulation of pro-tumorigenic chemokines CXCL1, CXCL3, CXCL5, and CCL20, along with corresponding chemokine receptors CCR1, CCR3, CCR4, CCR6, CCR8, and CCR10 that were positively correlated with the risk score. Such chemokine–receptor interactions can promote immunosuppression and immune evasion, thereby facilitating tumor progression. However, given the complexity of chemokine networks, no single chemokine could fully define the immunological role of the risk signature within the TME.

The high-risk group also showed higher levels of several immune cell types, including CD4^+^ T cells, dendritic cells, NK/T cells, T helper 2 cells, and MDSCs, compared to the low-risk group (all P < 0.001, **Figure 6B**). The risk score was positively correlated with the proportions of MDSCs (R = 0.20), regulatory T cells (R = 0.15), CD4^+^ T cells (R = 0.34), and activated dendritic cells (R = 0.25) (**Figures 6C–F**). Consistent with an immune-excluded or immunosuppressive microenvironment, and PD-1 expressions, typically low in non-inflamed tumors, were relatively high in the high-risk group.

In line with these observations, numerous immune-related genes were differentially expressed in correlation with the risk score (**Figures 6G, H**). While no single immune marker showed a perfect linear relationship with the risk score, several inhibitory immune checkpoint genes and markers, including CD276, LAIR1, HAVCR2, CD80, and CD86, were significantly associated with higher risk scores. This further supports the presence of an immunosuppressive milieu in high-risk patients. Notably, the checkpoint receptors PDCD1 and TIGIT, which are indicative of T-cell exhaustion, were more highly expressed in the high-risk group.

### Identification of therapeutic targets and drugs for high-risk HCC

**Figure 7:**
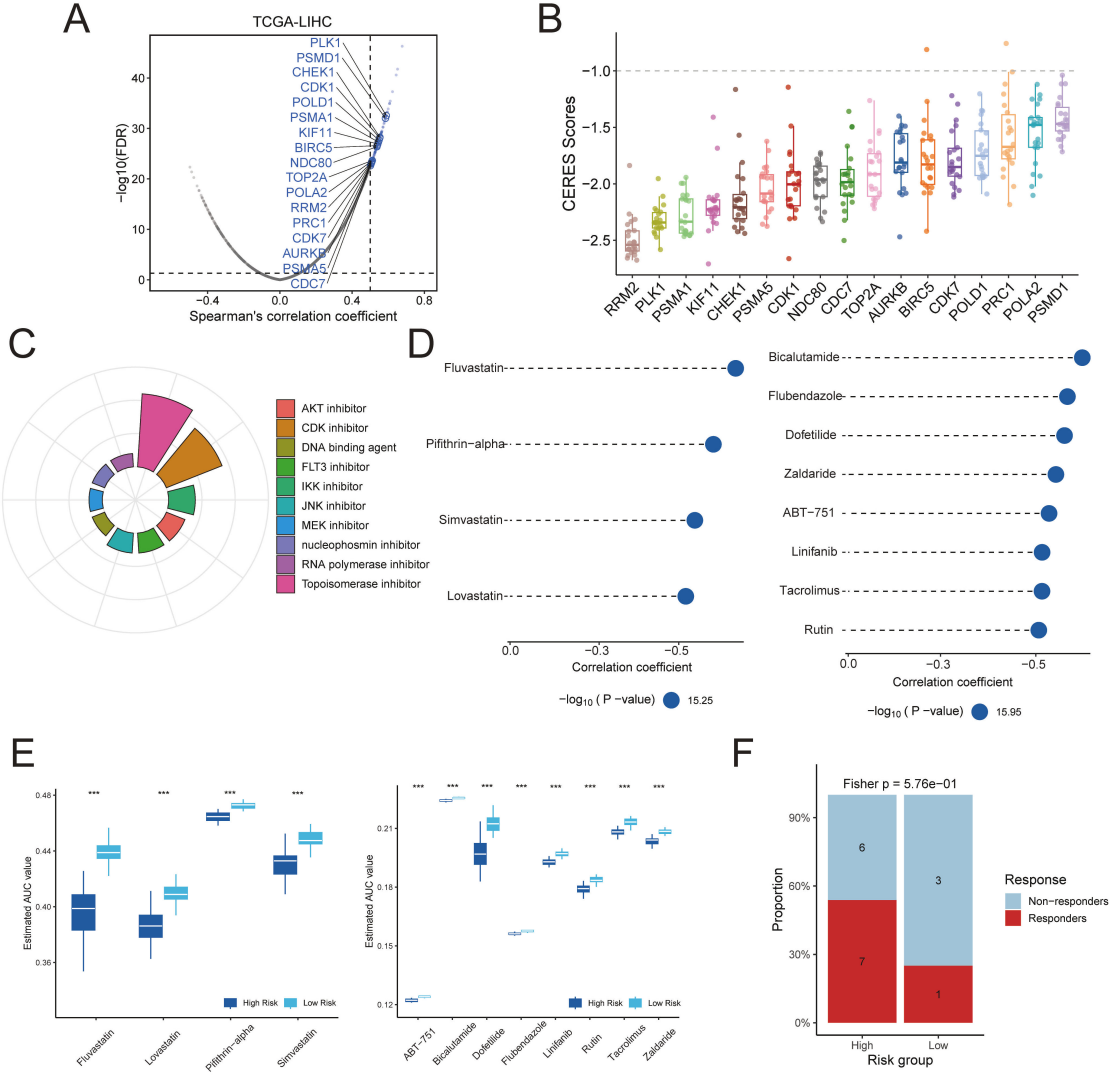
Identification of potential therapeutic targets and agents for TPS-high patients was conducted. **(A)** Dot plots illustrate the correlation coefficients derived from Spearman’s rank correlation analysis between TPS and druggable mRNA expression in the TCGA-LIHC datasets. Light blue dots represent potential targets that meet the threshold in Spearman’s rank correlation analysis (R > 0.3, adjusted P < 0.05), while dark blue dots indicate targets also selected by CERES analysis. **(B)** The distribution of CERES scores for identified targets in HCC cell lines is shown. **(C)** The composition of chemical compounds identified by Connectivity Map (CMap) analysis is presented, with only the top 10 drug categories displayed. **(D)** Drug sensitivity predictions for the high-risk TPS group in the TCGA hepatocellular carcinoma cohort were performed based on data from the CTRP and PRISM databases. **(E)** The inferred area under the curve (AUC) values of potential drugs from CTRP and PRISM databases were compared between TPS-high and TPS-low patients in the TCGA-LIHC dataset. **(F)** Assessment of immunotherapy response in the TPS high-risk subgroup using the HCC immunotherapy dataset GSE202069.

To uncover potential therapeutic targets linked to the risk signature, we correlated the risk score with the expression of druggable genes. A Spearman’s rank correlation analysis in the TCGA-LIHC cohort identified 115 genes whose expression was positively correlated with the risk score (R > 0.5, FDR < 0.05 in both the training and validation sets) (**Figure 7**, **Supplementary Tables S10**, **S11**). We considered these genes as candidate targets associated with the high-risk state. To assess their functional importance, we examined CERES dependency scores in 22 HCC cell lines. This analysis narrowed the list to 17 high-priority targets that showed strong genetic dependency (CERES scores predominantly below –1) (**Figure 7**, **Supplementary Tables S14**, **S15**). Many of these candidate targets, for example, CDK7, PRC1, PLK1, CDK1, BIRC5, CDC7, KIF11, NDC80, and AURKB, are critical regulators of cell division, aligning with the proliferative nature of high-risk tumors.

Next, we used the Connectivity Map (CMap) to predict drugs that could reverse the high-risk gene expression profile. We first derived a consensus set of differentially expressed genes (DEGs) that characterize the high-risk tumors. Using a meta-analysis of expression data, we identified 2,543 DEGs distinguishing high-risk from low-risk cases (**Supplementary Table S16**). We selected the top 150 upregulated and top 150 downregulated genes from this list to serve as a representative “signature” of high-risk tumors. This 300-gene expression signature was queried against the CMap database. As a result, we identified 84 small-molecule compounds with CMap connectivity scores below –90, indicating a strong predicted ability to invert the high-risk transcriptional program (**Figure 7**). Among these candidate compounds, approximately 13.1% were topoisomerase inhibitors and 10.7% were CDK inhibitors.

We further prioritized therapeutic compounds by integrating drug sensitivity data from PRISM and CTRP databases. Predicted drug AUC values were compared between high-risk and low-risk groups for the top CMap candidates. Twelve compounds were identified that showed significantly greater efficacy in high-risk patients from the TCGA-LIHC cohort (**Figure 7D**). According to the CTRP data, high-risk tumors were more sensitive to HMG-CoA reductase inhibitors (e.g., fluvastatin, simvastatin, lovastatin) and to the p53 inhibitor pifithrin-α. Likewise, analysis of PRISM data suggested that other agents, including bicalutamide, flubendazole, dofetilide, zaldaride, AB-751, linifanib, tacrolimus, and rutin, could be selectively effective in high-risk HCC cases. Additionally, The result of HCC immunotherapy cohort demonstrated that patients in the high-risk group exhibited a significantly better response to immunotherapy. (**Figure 7**, **Supplementary Figure S3**).

### The landscape of crosstalk between tumor cells and immune cells

**Figure 8:**
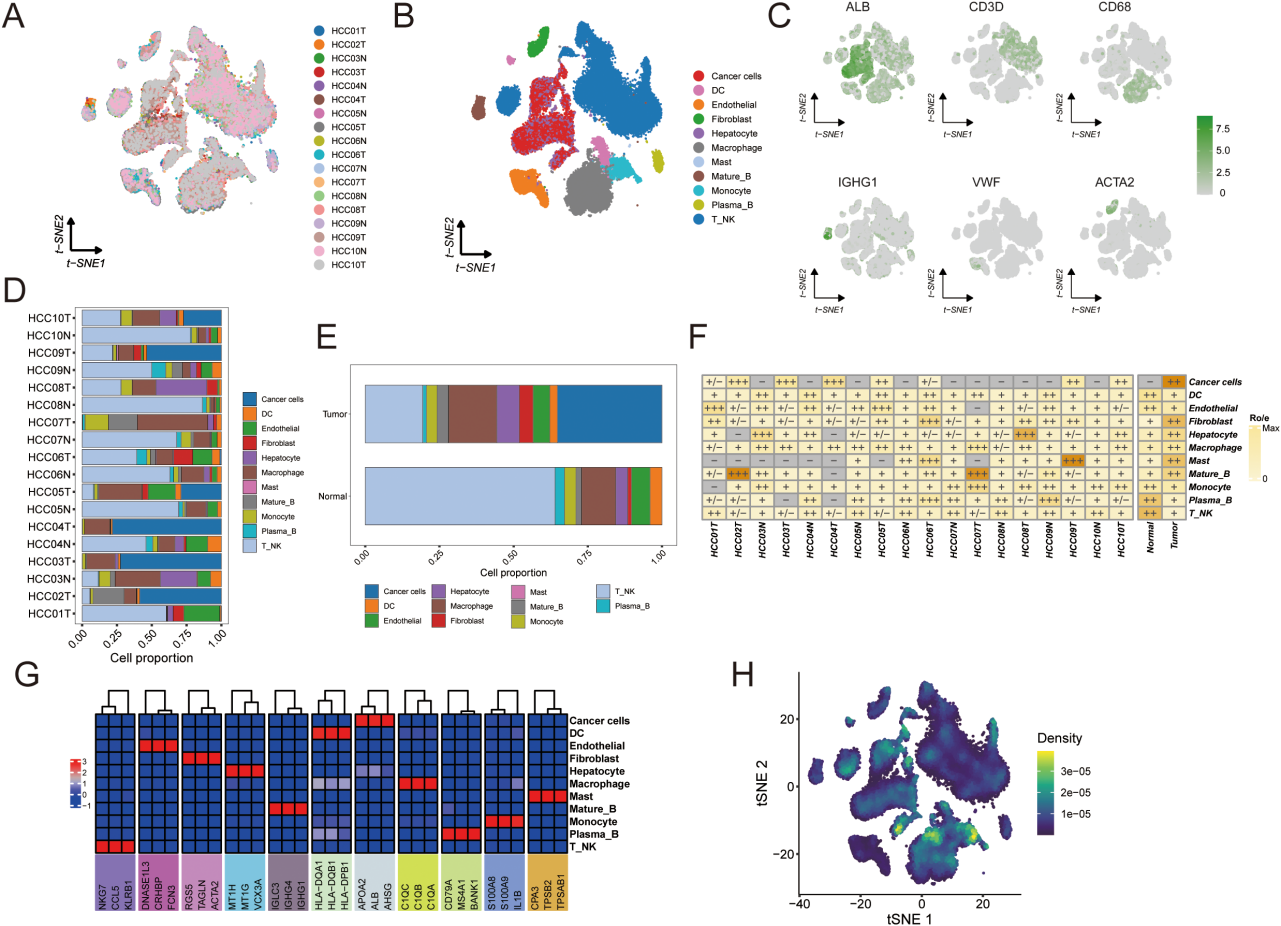
Single-cell analysis to identify tumor and normal subpopulations in HCC. The visualizations and analyses provide insights into cellular diversity and marker expression patterns: **(A)** tSNE visualization displaying cells from 10 HCC patients. **(B)** identification of subclusters from 10 HCC patients. **(C)** The tSNE plots illustrate the expression patterns of signature genes across six primary cell types, with color gradients representing gene expression levels. **(D)** The proportions of major cells in each sample. **(E)** The proportions of major cells across all samples. **(F)** Tissue preference of each cell type in each sample estimated by Ro/e. **(G)** Heatmap showing the presence of different cell subsets. **(H)** tSNE visualization based on TPS signature enrichment across cellular subsets.

To gain insight into cell–cell interactions in the tumor microenvironment, we analyzed scRNA-seq data from 10 HCC patients. After stringent quality control, we obtained a dataset of 58,896 cells from primary tumors, metastatic tumors, and normal liver tissue. Unsupervised clustering identified 38 distinct cell clusters, and their stability was confirmed using down-sampling and leave-one-patient-out validation analyses. We classified these clusters into 10 major cell types based on canonical marker genes (**Figures 8A–C**, **Supplementary Tables S17**, **S19**).

Tumor tissues displayed a highly heterogeneous cellular composition with an abundance of immune and stromal cells. Macrophages, dendritic cells (DCs), fibroblasts, and T/NK cells were more prominent in tumors, reflecting an inflammatory microenvironment (**Figures 8D, E**). In contrast, normal liver tissues were predominantly composed of hepatocytes with far fewer immune cells. Notably, both tumor and normal samples contained mature B cells and plasma B cells, although their frequencies were slightly higher in normal tissue. Endothelial cells were present in both settings but showed greater variability among normal samples. These observations indicate that HCC tumors harbor significantly more immune infiltrates and stromal components than adjacent normal liver, underscoring the immunologically active nature of the tumor microenvironment.

We further explored inter-patient heterogeneity by analyzing the tissue preference of each cell type using a Ro/e metric (**Figure 8F**). Unique marker genes were identified for all 10 cell types based on differentially expressed genes. **Figure 8G** illustrates the cellular composition of each sample annotated by these marker genes, revealing several noteworthy patterns. For instance, patients whose tumors had high expression of NKG7 in T/NK cells exhibited robust anti-tumor immune activity characterized by an expansion of cytotoxic lymphocytes. Tumors with elevated S100A8 and S100A9 expression showed a pronounced expansion of myeloid cell populations, indicative of an immunosuppressive phenotype. Additionally, samples with high levels of KRT8 and KRT18 contained an abundance of malignant hepatocytes (**Figure 8**). To visualize where the TAM polarity signature is active within the tumor, we generated a t-SNE plot mapping the enrichment of the TPS gene signature across different cell populations (**Figure 8H**).

**Figure 9:**
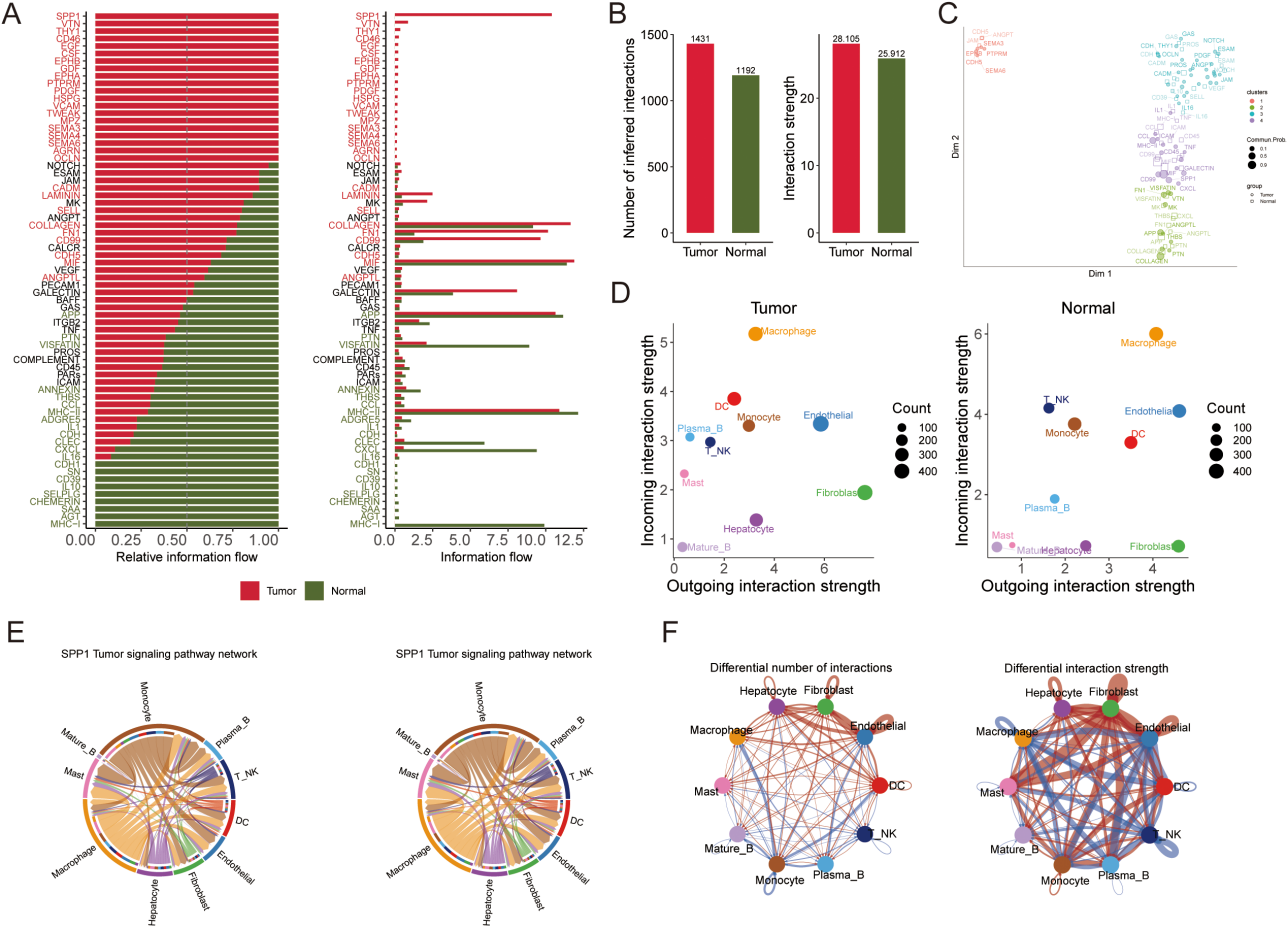
The results of the intercellular communication analysis and the SPP1 signaling pathway are summarized, which illustrate potential incoming and outgoing signaling pathways among various cell types. **(A)** The intercellular communication quantity and strength of tumor and normal tissue. **(B)** The differential number and intensity of cell-cell interactions between cells in HCC. **(C)** Bubble diagram visualizing the overall profile of possible incoming or outgoing signaling pathways between cells in HCC. **(D)** The relative and actual information flow of tumor and normal tissue based on ligand-receptor interactions. **(E)** The intensity of cell-cell interactions in the SPP1 signaling pathway in different cell subpopulations. **(F)** The differential number and strength of cellular interactions.

We next examined cell–cell communication networks in the tumor versus normal single-cell datasets. Using ligand–receptor interaction analysis, we found that intercellular communication was globally more extensive and stronger in tumor tissue compared to normal tissue (**Figures 9A, B**). Tumor endothelial cells were identified as central signal senders and receivers in the network, highlighting their key role in connecting with multiple cell types. Interestingly, communication between macrophages and other immune cells was reduced in tumors relative to normal tissue (**Figures 9C, D**), suggesting that macrophage interactions in the tumor are altered and may influence immune cell infiltration.

Pathway analysis of the differential ligand–receptor interactions between tumor and normal environments highlighted SPP1 as a significantly upregulated signaling factor in tumors. We analyzed the expression of SPP1 and its receptor-associated genes across the cell types in primary tumor, metastatic, and normal samples (**Figure 9E**). SPP1 emerged as a central mediator of cell–cell crosstalk in the tumor microenvironment, with especially high signaling activity between macrophages and various immune cells (monocytes, plasma B cells, T/NK cells, DCs) (**Figure 9F**). This suggests that SPP1-driven interactions play a crucial role in shaping the immune microenvironment in HCC.

### Validation of TAM polarity-related signature expression in HCC patients

**Figure 10:**
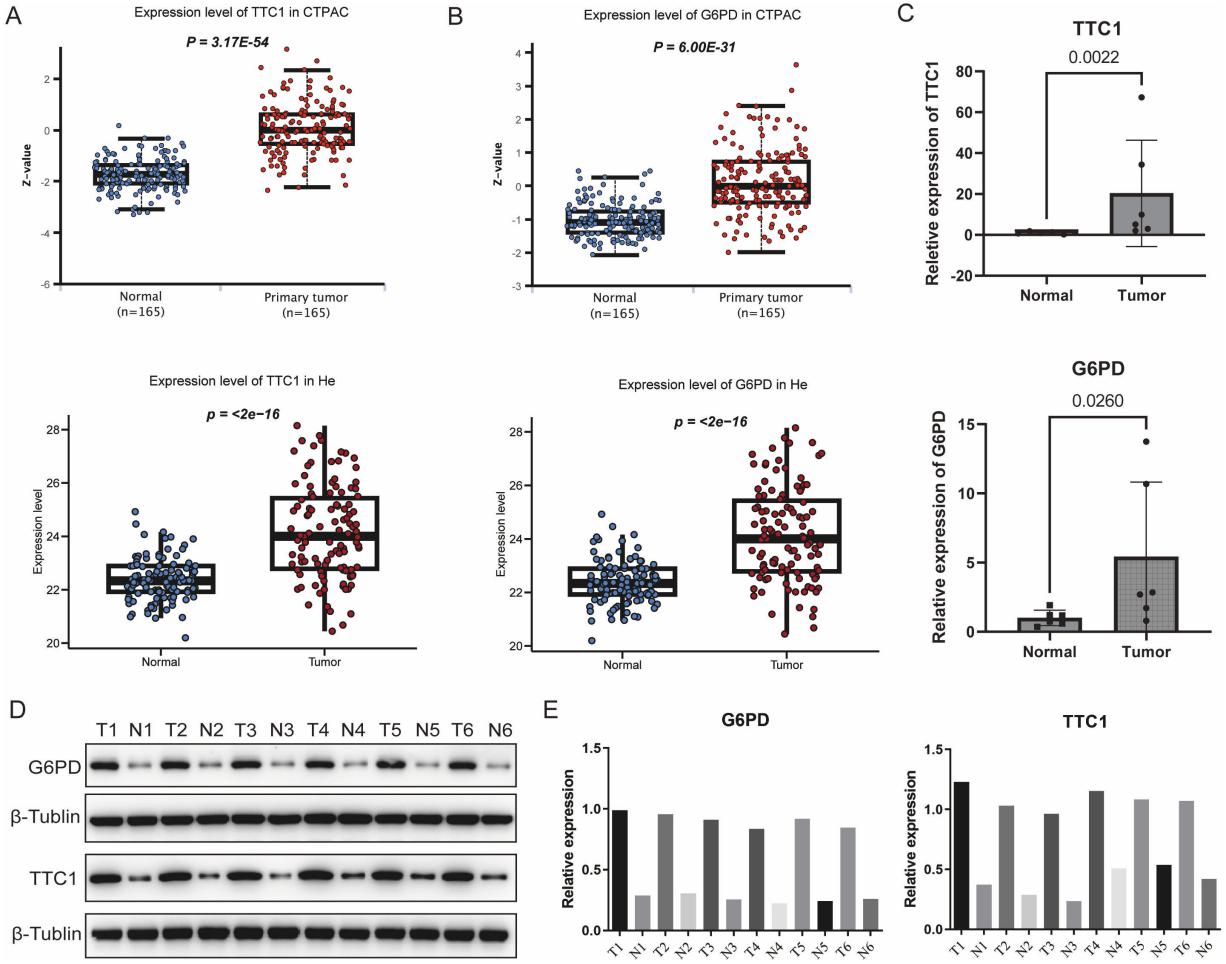
Validation of the expression levels of signature genes. **(A)** Comparative analysis of TTC1 and G6PD expression between HCC and normal liver tissues using integrated data from TCGA database. **(B)** Expression profiles of TTC1 and G6PD in normal versus tumor tissues were further analyzed using the UALCAN database. **(C)** Quantitative reverse transcription PCR results further confirmed significant differential expression of TTC1 and G6PD between HCC and normal tissues (n=6 pairs). **(D, E)** Validation of TTC1 and G6PD protein expression levels in HCC tumor samples and paired adjacent non-tumor tissues by Western blot (n=6 pairs).

Notably, TTC1 and G6PD exhibited significantly higher expression levels in tumor cells compared to other cell types within the TME. Furthermore, a machine learning-based feature importance analysis identified TTC1 and G6PD as the top two most influential genes in the prognostic model, leading to their selection for experimental validation (**Supplementary Figure S3**). These genes were prioritized for validation because public transcriptomic and proteomic datasets indicated that both are highly upregulated in tumors compared to normal liver (**Figures 10A, B**). We first confirmed their overexpression in clinical tissue samples by performing qRT-PCR and Western blot on 10 pairs of HCC tumors and adjacent non-tumor liver tissues. Consistent with the database predictions, TTC1 and G6PD mRNA levels were markedly higher in tumor tissues than in matched normal tissues, which mirrors their elevated protein levels in tumors (**Figures 10C–E**, **Supplementary Figures S6**, **S7**).

**Figure 11:**
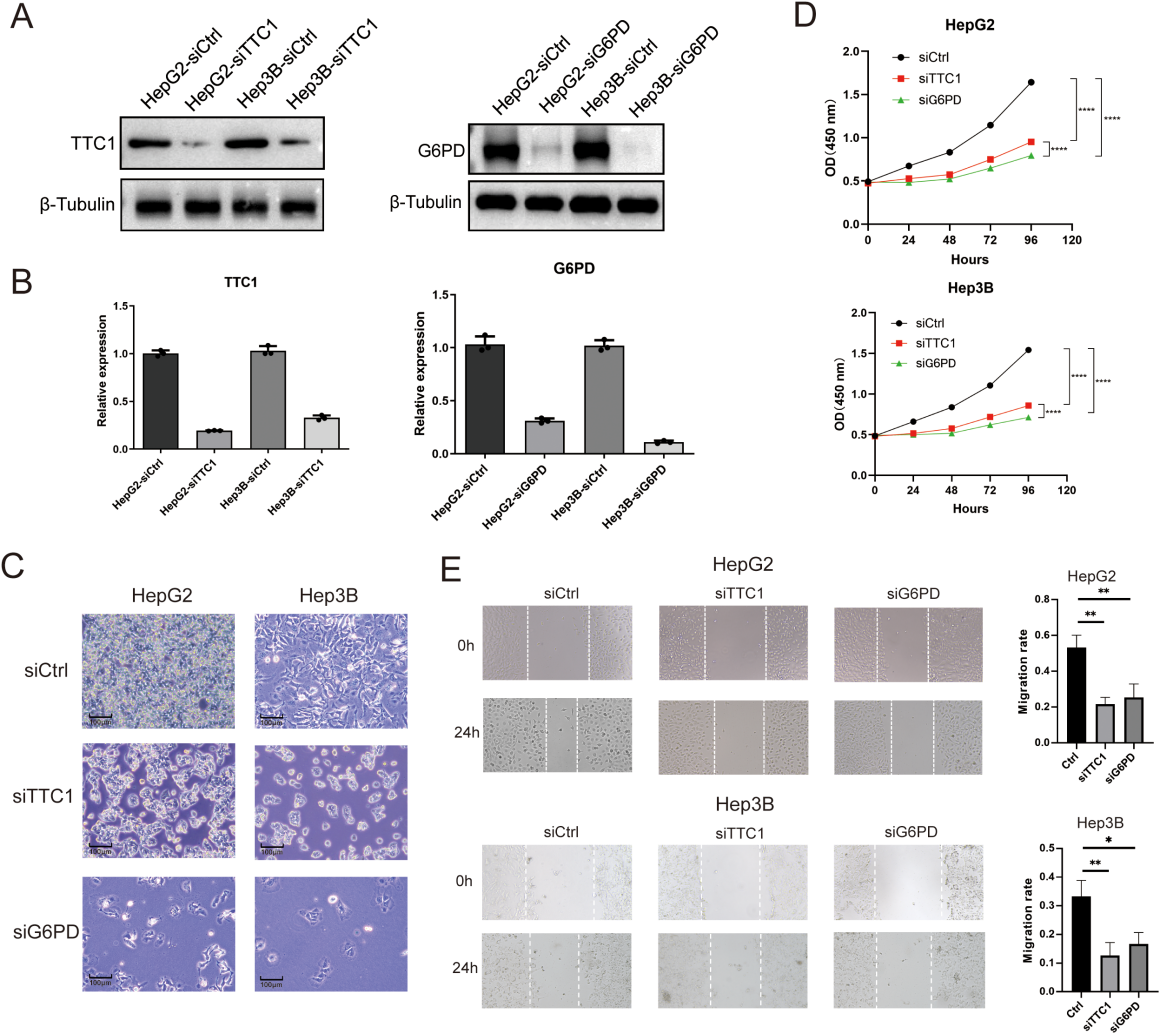
Knockdown of TTC1 and G6PD suppresses cell proliferation. **(A, B)** Western blot analysis showing reduced expression of TTC1 and G6PD in HepG2 and Hep3B cells following siRNA-mediated knockdown. **(C)** Depletion of TTC1 and G6PD via RNAi reduces cell proliferation, as observed by live-cell imaging. **(D)** Cell proliferation was assessed by CCK-8 in control, TTC1-knockdown, and G6PD-knockdown groups (n=3). **(E)** Wound healing assay demonstrates that downregulation of TTC1 and G6PD expression reduces the migration ability of HepG2 and Hep3B cell lines. (* p < 0.05, ** p < 0.01, *** p < 0.001).

We next investigated the effects of TTC1 and G6PD knockdown in HCC cell lines. Using siRNA, we silenced each gene in HepG2 and Hep3B cells, achieving efficient knockdown as confirmed by reduced mRNA and protein expression (**Figure 11A**, **Supplementary Figures S8**, **S9**). Loss of TTC1 or G6PD significantly suppressed the proliferation of both HCC cell lines (P < 0.05; **Figure 11C**). In wound-healing assays, cells with TTC1 or G6PD knockdown showed a significantly wider scratch gap at 24 hours compared to control cells (P < 0.05; **Figure 11**, **Supplementary Figures S10**, **S11**). This indicates that silencing either gene markedly impairs the migratory ability of HCC cells *in vitro*.

## Discussion

Tumor-associated macrophages (TAMs), a predominant component of the TME, play a pivotal role in the progression and in shaping the immune landscape of various cancers, including HCC (20). Macrophage polarization, often classified into pro-inflammatory M1 and anti-inflammatory M2 phenotypes, has been shown to significantly influence tumor progression, immunosuppression, and response to therapy (21, 22). Also, previous studies have explored the biological function of TAMs in the TME and often treating macrophage biology as just one component of a larger immune mixture. Because the balance between classically activated (M1-like) and alternatively activated (M2-like) phenotypes directly shapes antigen presentation, T-cell recruitment, and immunosuppression, a polarity-focused model may provide complementary information (18, 23). Recently, Ruben et al. identified a novel macrophage polarity signature defined by CXCL9 and SPP1, termed CS-macrophage polarity, which demonstrated a strong association with cancer prognosis and was integrated within a broader network of tumor promoting or suppressing cellular interactions (9). As reported, SPP1 acts as a potent oncogene that enhances HCC cell proliferation, migration, invasion, and confers resistance to therapy. A key mechanism identified is its role in activating the fatty acid metabolic pathway, which provides energy and building blocks for rapidly dividing tumor cells. Inhibition of fatty acid oxidation has been shown to reverse these pro-tumorigenic effects, highlighting the SPP1-fatty acid metabolism axis as a crucial vulnerability in HCC (24). Beyond cell-autonomous effects, SPP1 is a pivotal component of immunosuppressive TME. Single-cell and spatial transcriptomic analyses have revealed that SPP1 is highly expressed by a specific subset of SPP1+ tumor-associated macrophages. These TAMs interact with cancer-associated fibroblasts via ligand-receptor pairs to stimulate extracellular matrix remodeling (25). This interaction forms a physical “tumor immune barrier” at the tumor boundary, which restricts the infiltration of cytotoxic CD8^+^ T cells into the tumor core, thereby creating an immune-excluded phenotype and contributing to resistance against immune checkpoint blockade therapy (26).

In this study, we developed a tumor-associated macrophage polarity signature to quantify macrophage infiltration and predict clinical outcomes in HCC using data from three independent cohorts. We benchmarked seven machine learning algorithms to construct a robust prognostic model, identifying the optimal classifier through comparative performance evaluation. Utilizing single-cell RNA sequencing data, we further explored intercellular communication by analyzing receptor-ligand interactions and signaling pathway alterations between tumor and normal tissues. Notably, SPP1-associated signaling pathways were markedly dysregulated in tumor tissues, underscoring the importance of TPS-driven signaling networks in shaping the HCC immune microenvironment. These results indicate that TPS and SPP1/CXCL9 play a crucial role in modulating tumor microenvironment in HCC. We also validated TTC1, PANK2 and G6PD expression levels in tumor by RT-qPCR. Furthermore, we explored whether TPS could guide therapeutic strategies for HCC using in silico approaches, revealing that patients with high TPS were more likely to benefit from topoisomerase inhibitors. Additionally, we identified 17 potential therapeutic targets, such as PRC1, POLA2, and PSMD1, along with 11 therapeutic agents. In summary, this study introduced an innovative and reliable TPS that quantifies replication stress, predicts prognosis, and informs therapeutic decisions in HCC.

Machine learning has been increasingly employed to predict survival outcomes in oncology research (27, 28). However, the successful translation of these approaches into clinical practice remains a considerable challenge. The models utilized in our study were specifically designed to handle heterogeneous, high-dimensional datasets and are well-suited for survival analysis (29, 30). Penalized regression methods—such as Lasso, Ridge, and Elastic Net—effectively address multicollinearity and are particularly advantageous when the number of predictors far exceeds the number of observations, as they prevent overfitting through regularization (29, 30). Unlike Ridge regression, Lasso and Elastic Net perform variable selection by shrinking some coefficients to zero, enhancing model interpretability (31). Nevertheless, in the presence of highly correlated predictors, Lasso may fail to identify the most informative variables, whereas Elastic Net can assign similar weights to correlated features, thereby improving robustness (32). We also incorporated boosting-based approaches, including CoxBoost and XGBoost, which are designed to minimize prediction errors by combining multiple weak learners into a strong ensemble model. CoxBoost utilizes likelihood-based boosting for estimating the regression coefficient vector and allows for the incorporation of clinically relevant mandatory variables into the final model (33, 34). XGBoost, introduced by Chen et al. in 2016 (35), offers a computationally efficient and flexible gradient boosting framework that supports extensive hyperparameter tuning and regularization. These characteristics likely contribute to its superior predictive performance observed in our benchmarking. In addition, dimension-reduction methods such as SuperPC and plsRcox were evaluated. SuperPC identifies genes most correlated with survival outcomes and derives principal components from them to predict prognosis (36). However, interpretation of these components can be complex due to their multigene composition. Similarly, plsRcox applies partial least squares regression to construct Cox models, generating latent variables from linear combinations of all predictors (37). This can compromise model interpretability and potentially introduce noise, as irrelevant predictors may still contribute to the components (38). These limitations may account for the relatively lower predictive performance of these methods in our study. Notably, the performance of machine learning algorithms can be influenced by the characteristics of the training dataset, including sample size, feature distribution, and biological variability. Immunological profiling revealed that high-risk patients exhibited significantly elevated levels of regulatory T cells (Tregs) and myeloid-derived suppressor cells, indicative of an immunosuppressive tumor microenvironment (39). This phenotype may underlie the limited efficacy of immune checkpoint inhibitors as monotherapy in these individuals. Consequently, combination therapies, such as anti-PD-1 antibodies administered alongside Treg-depleting agents or MDSC-targeted interventions, may prove more effective in enhancing anti-tumor immunity. These strategies hold promise for reprogramming the immune microenvironment and improving treatment outcomes.

In this study, we constructed a prognostic signature based on CXCL9:SPP1 macrophage polarity using machine learning techniques. This signature demonstrated strong and consistent prognostic power across external HCC cohorts. To gain a deeper understanding of molecular differences, we performed integrative multi-omics analyses, which revealed substantial disparities between high- and low-risk groups in terms of genomic mutations, immune cell composition, and intercellular communication. These findings underscore the critical role of macrophage polarization in shaping the tumor immune microenvironment, thereby influencing both HCC progression and response to therapy.

Our results also suggest potential roles for TTC1, PANK2, and G6PD in the development and progression of HCC. TTC1, which has been implicated in cell cycle regulation and apoptosis, was significantly overexpressed in tumor tissues relative to adjacent normal tissues, suggesting that it plays a vital role in HCC pathogenesis (40). Further bioinformatic analysis indicated that TTC1 may act as a downstream effector of p62, an oncogenic scaffold protein involved in the dysregulation of multiple liver cancer-related genes (41). Given its association with disease severity, TTC1 holds promise not only as a prognostic biomarker but also as a potential therapeutic target. PANK2, the sole mitochondrial isoform of pantothenate kinase, is markedly upregulated in HCC tissues. It has been associated with metabolic reprogramming, including enhanced lipid metabolism and energy production. Garcia et al. reported that PANK2 contributes to the dysregulation of metabolic pathways, thereby promoting tumor cell proliferation and malignancy (42). Furthermore, PANK2 is involved in signaling pathways related to mitochondrial function and oxidative stress. Wang et al. also found that PANK2 expression correlates with clinical outcomes and immune infiltration in HCC (43). G6PD, encoding glucose-6-phosphate dehydrogenase, is a key enzyme in the pentose phosphate pathway and plays an essential role in maintaining redox homeostasis and anabolic metabolism in cancer cells. Its overexpression in HCC has been linked to disease progression, with elevated G6PD activity supporting NADPH production, oxidative stress resistance, and lipid biosynthesis (44). Moreover, G6PD has been shown to facilitate tumor migration and invasion through the induction of epithelial-mesenchymal transition, thereby promoting metastasis (45). Given its involvement in glucose metabolism and redox regulation, G6PD represents a potential prognostic biomarker and therapeutic target in HCC, with inhibitors of G6PD or the pentose phosphate pathway offering promising avenues for treatment.

This study presents a machine learning, based framework for evaluating and comparing multiple survival prediction models, leading to the identification of a TPS with superior prognostic performance across multiple cohorts. However, several limitations should be acknowledged. The absence of prospective validation cohorts and the lack of experimental functional validation for TPS related genes (including rescue experiments, multiplex immunofluorescence or spatial transcriptomics) may limit the immediate clinical applicability of our findings. Furthermore, heterogeneity in cohort characteristics may introduce bias Further investigation is necessary to fully elucidate the biological mechanisms and therapeutic relevance of TPS. This should include functional validation of key TPS genes using *in vitro* and *in vivo* models to clarify their roles in tumor progression and metastasis. Also, candidate inhibitors or molecular interventions should be tested in cell-based assays and patient-derived xenograft models to determine their anti-tumor efficacy. Combination strategies with existing therapies, such as immune checkpoint inhibitors, should also be explored to assess potential synergistic effects.

## Conclusion

In conclusion, this study developed a robust and effective prognostic model for HCC using multiple machine learning algorithms. The TPS demonstrates strong potential for clinical risk stratification and may inform personalized treatment strategies for HCC patients.

## Data Availability

The original contributions presented in the study are included in the article/**Supplementary Material**, further inquiries can be directed to the corresponding author/s.
