# Supplementary material for: Machine learning-based tumor associated macrophages polarity signature predicts prognosis and treatment response in hepatocellular carcinoma
Source: Front Immunol. 2025 Nov 5;16:1663519. doi: 10.3389/fimmu.2025.1663519 (PMC12627068; doi:10.3389/fimmu.2025.1663519)
Supplement: Supplementary file 2 [file Table2.docx]

**Supplementary figures:**

**
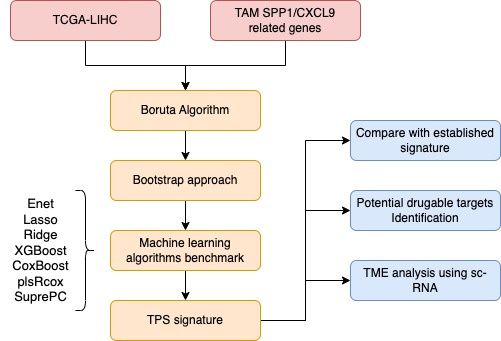
**

**Figure S1.** Schematic overview of the bioinformatics workflow for constructing and validating the TPS.

**
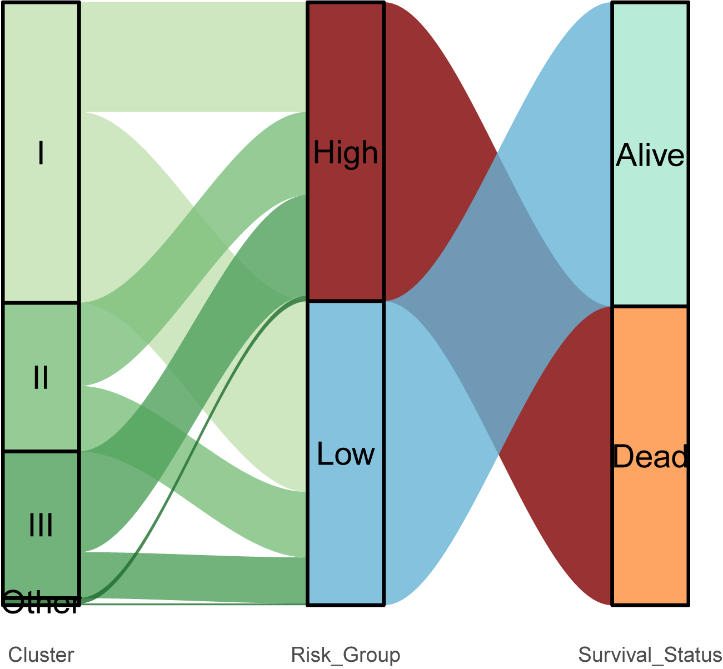
**

**Figure S2.** Sankey diagram illustrates the relationships among factors with significant differences in Cox regression analysis (tumor stage), TPS-based risk stratification, and patient prognosis.​

**
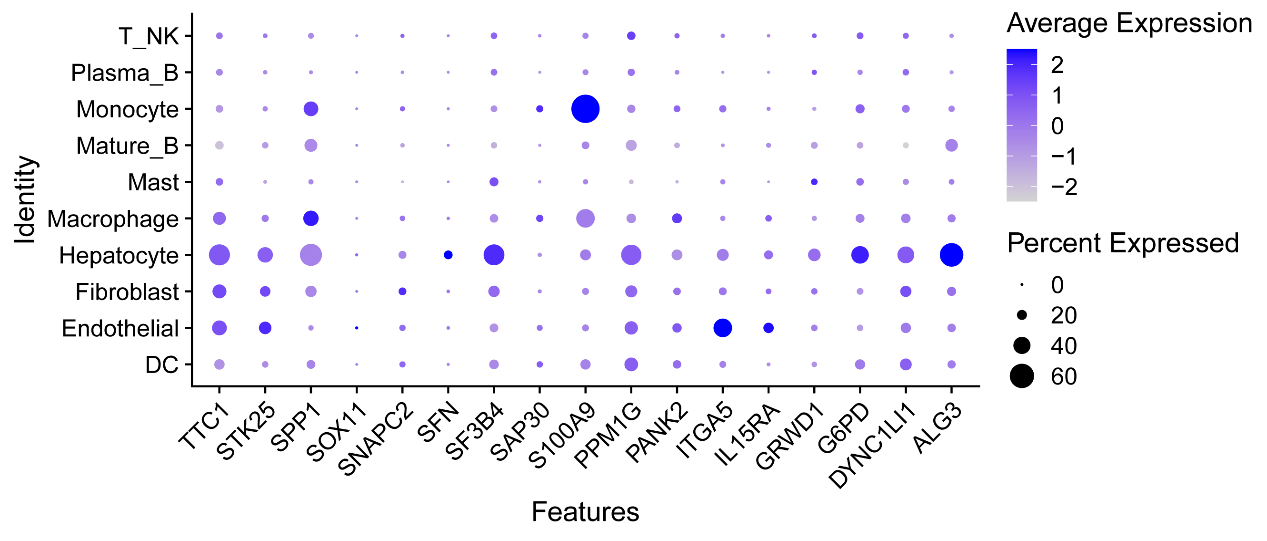
**

**Figure S3.** Expression of TPS signature genes across distinct cell subpopulations.

**
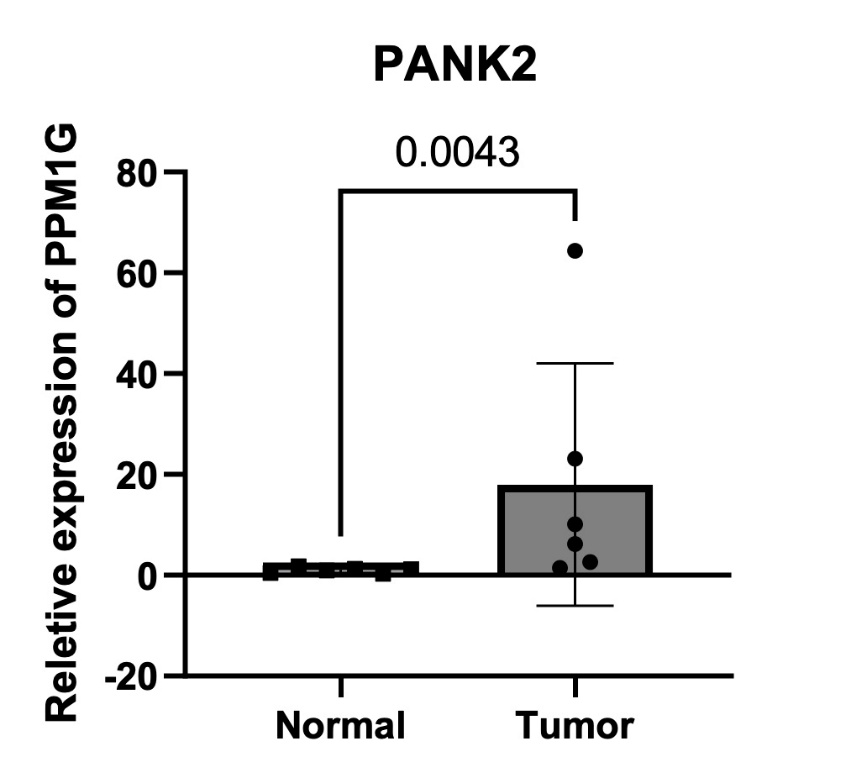
**

**Figure S4.** Quantitative reverse transcription PCR (qRT-PCR) results further confirmed significant differential expression of PANK2 between HCC and normal tissues (n = 6 pairs).

**
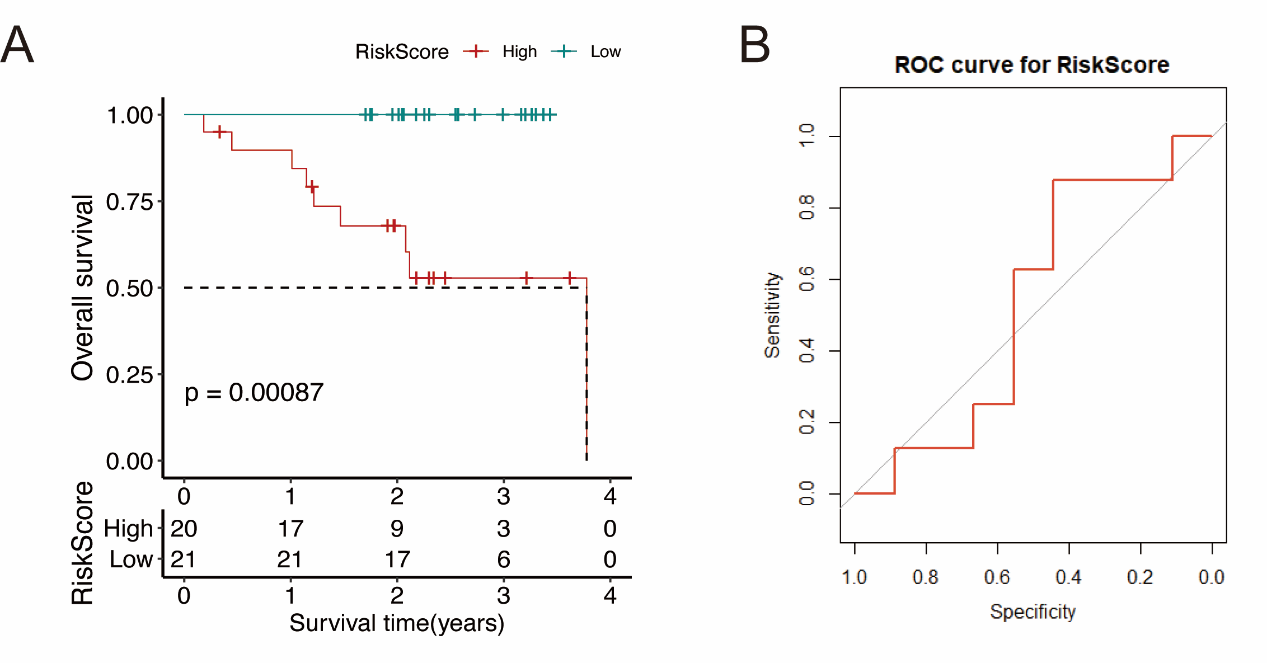
**

**Figure S5**. Assessment of the TPS in the HCC immunotherapy cohort GSE202069.​​ (A) Kaplan-Meier survival curves​ comparing overall survival between high-risk and low-risk groups stratified by the TPS. (B) ROC curve​ evaluating the predictive accuracy of the TPS for survival outcomes.


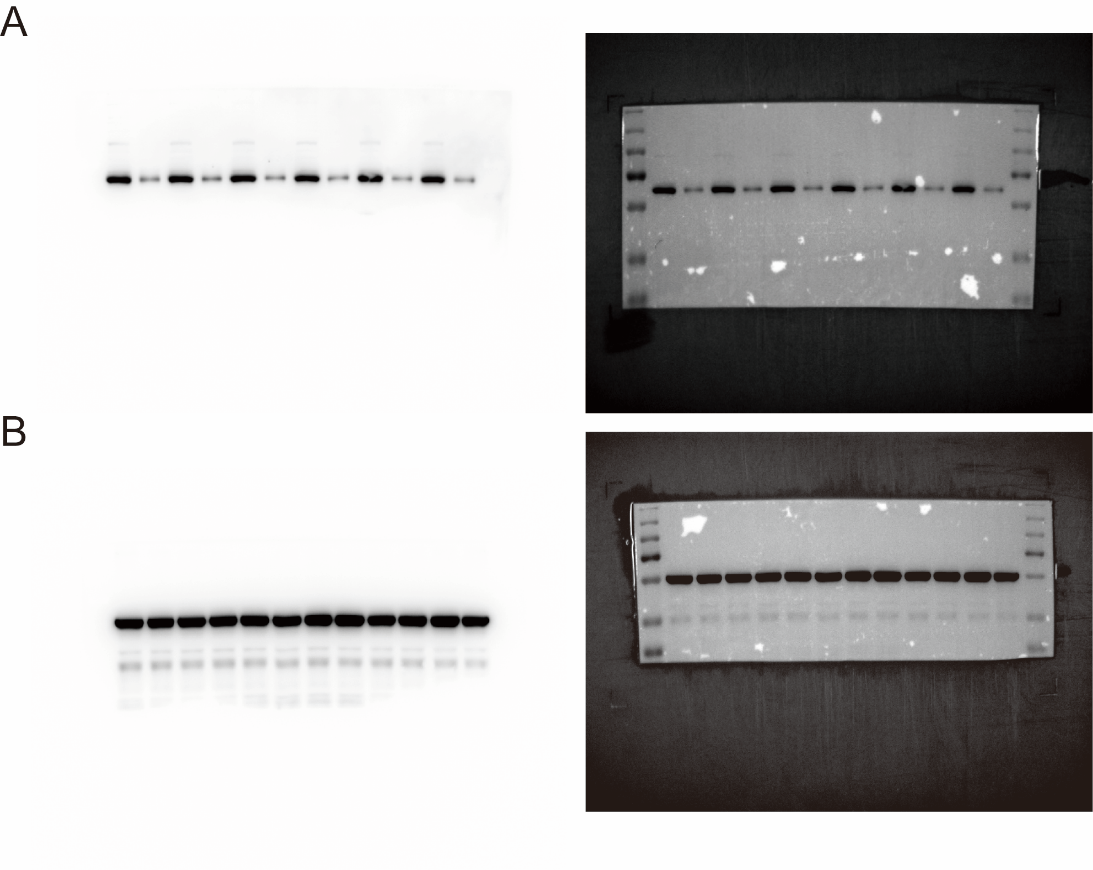


**Figure S6**. G6PD expression in HCC samples detected by WB original scan picture. (A) G6PD uncropped, exposure time 10 seconds. (B) Tubulin uncropped, exposure time 2 seconds.


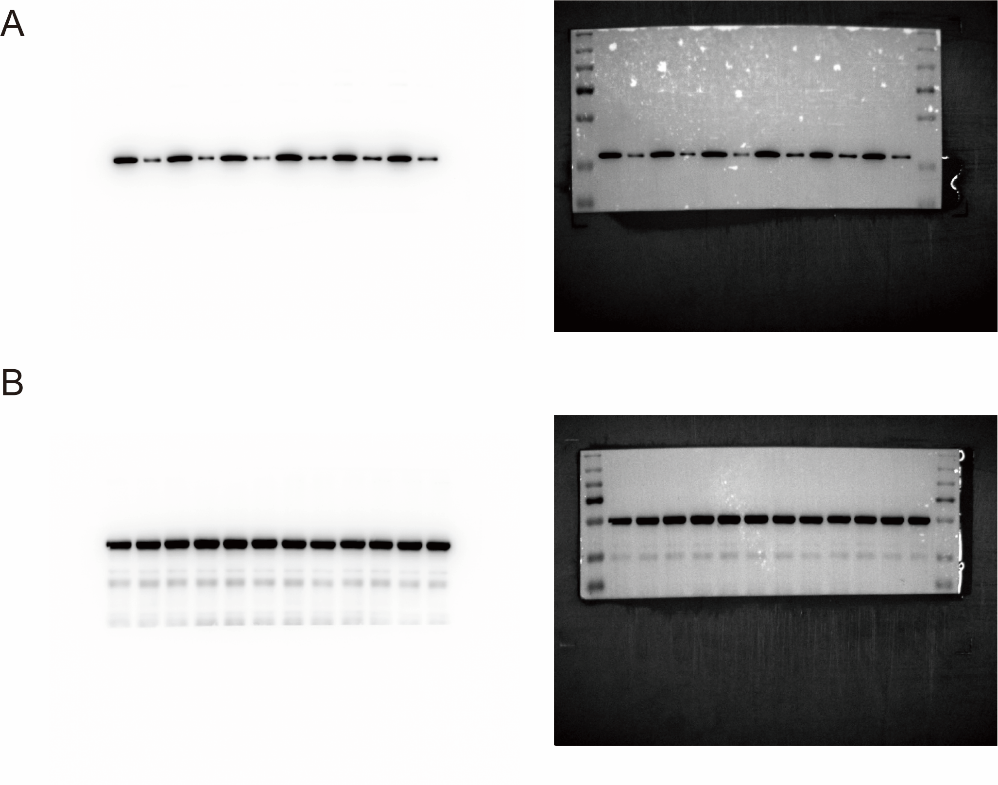


**Figure S7**. TTC1 expression in HCC samples detected by WB original scan picture. (A) TTC1 uncropped, exposure time 10 seconds. (B) Tubulin uncropped, exposure time 2 seconds.

**
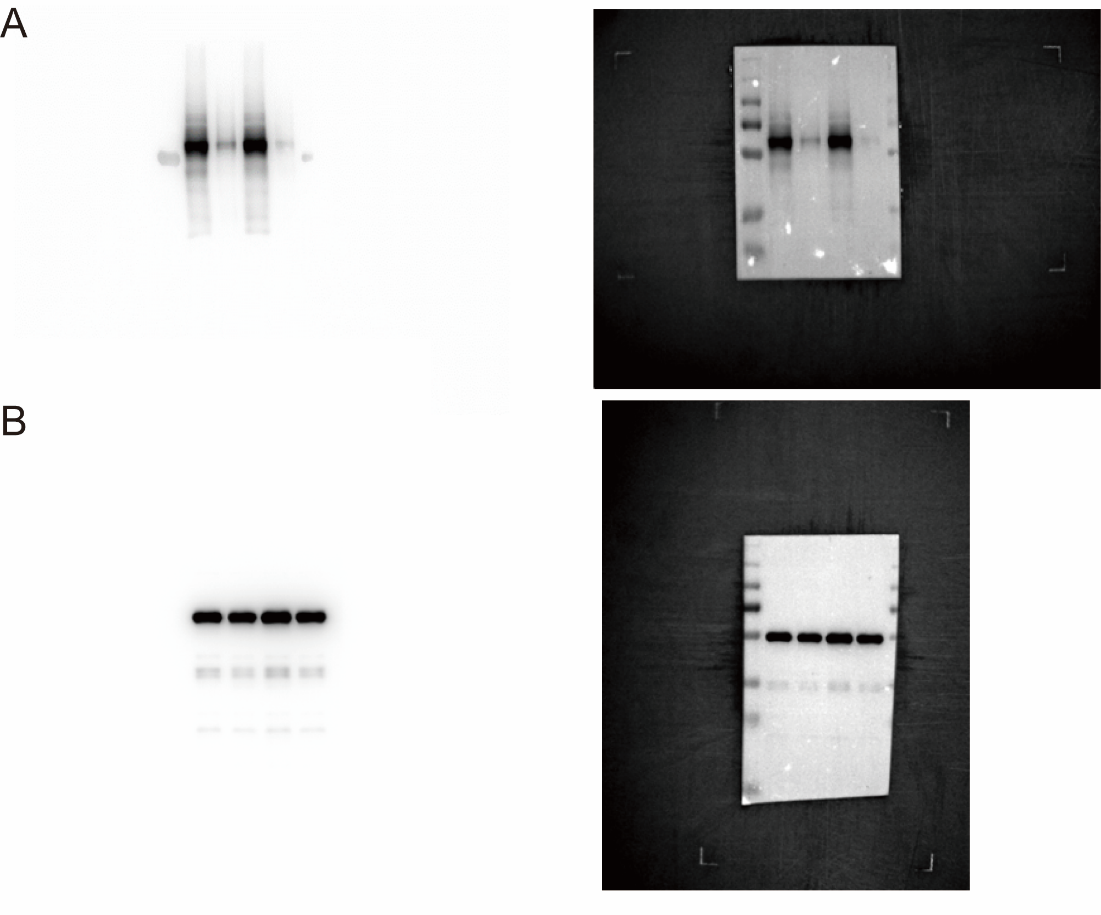
**

**Figure S8**. siG6PD WB original scan picture. (A) G6PD uncropped, exposure time 2 seconds. (B) Tubulin uncropped, exposure time 2 seconds.

**
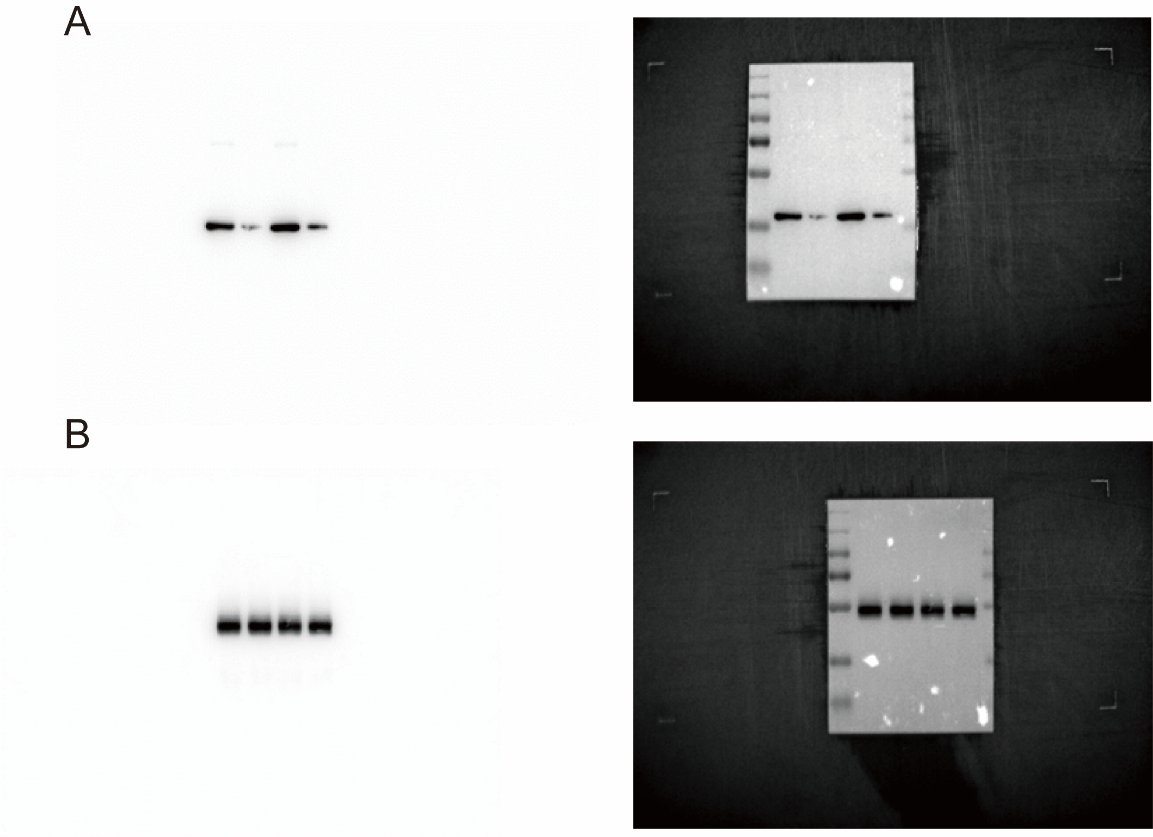
**

**Figure S9**. siTTC1 WB original scan picture. (A) TTC1 uncropped, exposure time 5 seconds. (B) Tubulin uncropped, exposure time 2 seconds.


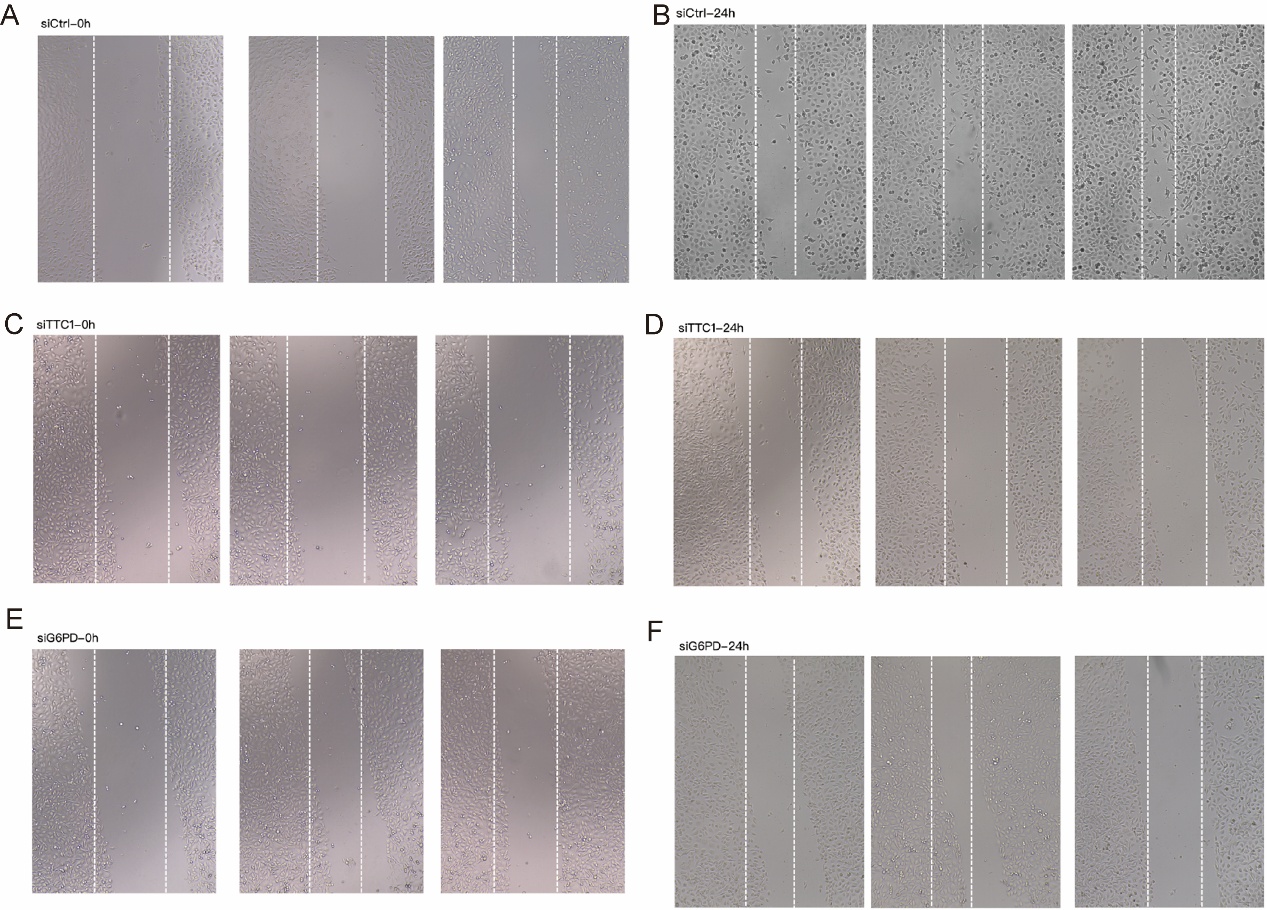


**Figure S10**. Wound healing assays in HepG2 cell line. (A) siCtrl group at 0 h. (B) siCtrl group at 24 h. (C) siTTC1 group at 0 h. (D) siTTC1 group at 24 h. (E) siG6PD group at 0 h. (F) siG6PD group at 24 h.


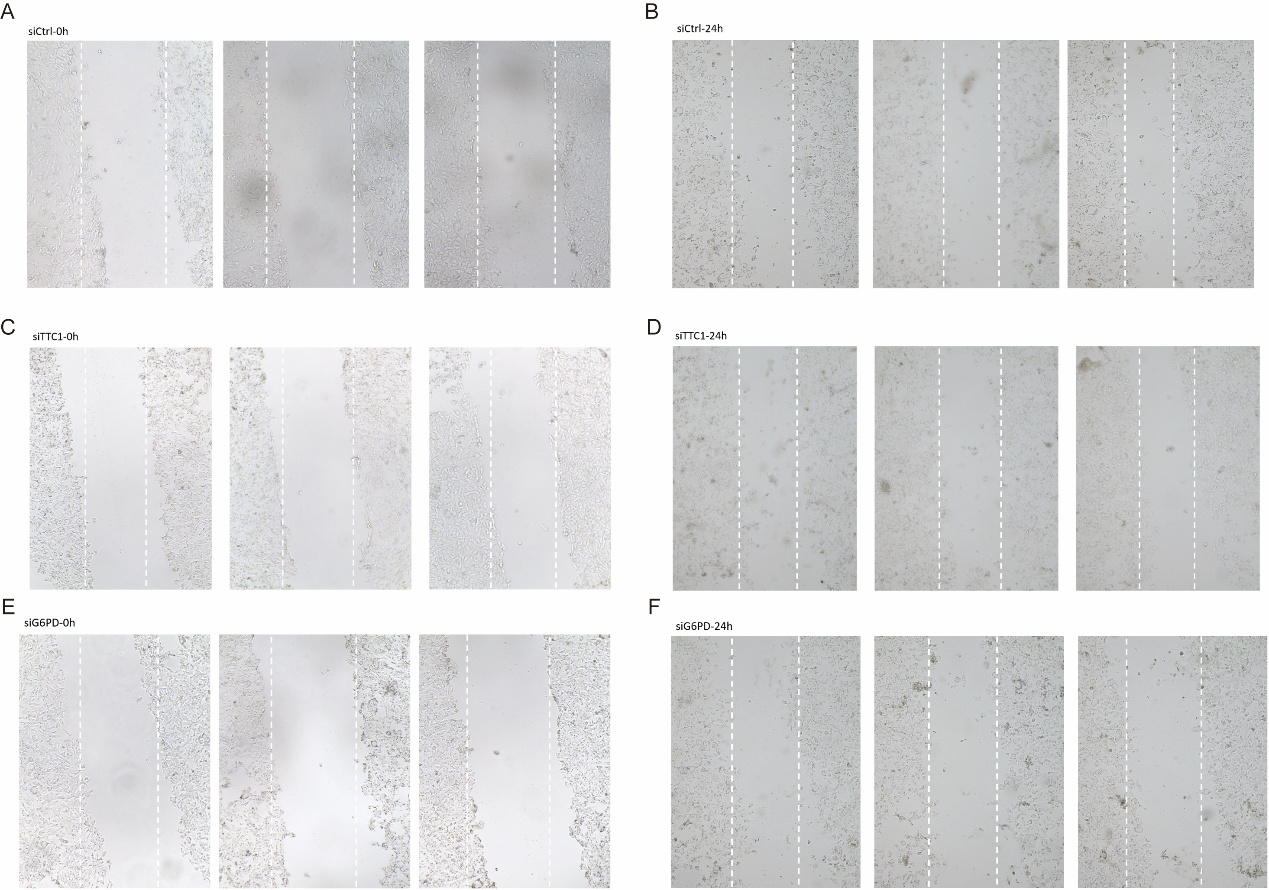


**Figure S11**. Wound healing assays in Hep3B cell line. (A) siCtrl group at 0 h. (B) siCtrl group at 24 h. (C) siTTC1 group at 0 h. (D) siTTC1 group at 24 h. (E) siG6PD group at 0 h. (F) siG6PD group at 24 h.
