# Supplementary figures and images for: Machine learning-based tumor associated macrophages polarity signature predicts prognosis and treatment response in hepatocellular carcinoma
Source: Front Immunol. 2025 Nov 5;16:1663519. doi: 10.3389/fimmu.2025.1663519 (PMC12627068; doi:10.3389/fimmu.2025.1663519)

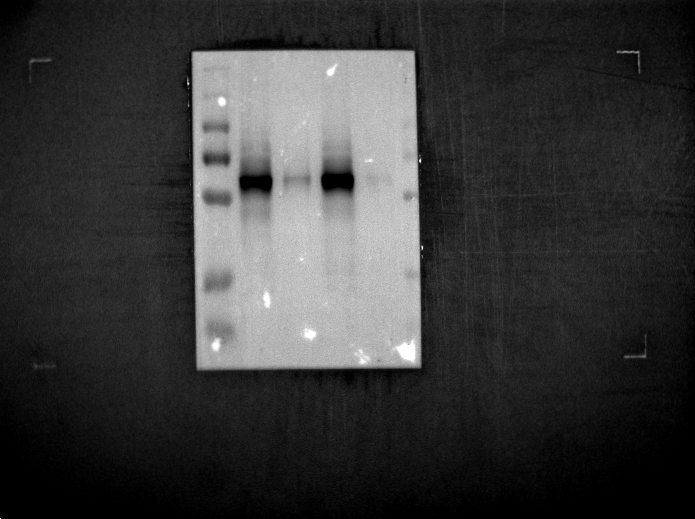

Supplement: Supplementary file 3 [file Image1.tif]

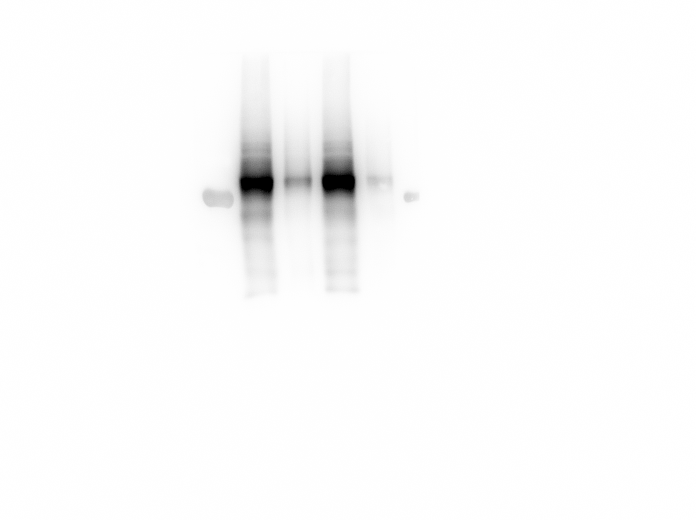

Supplement: Supplementary file 4 [file Image2.tif]

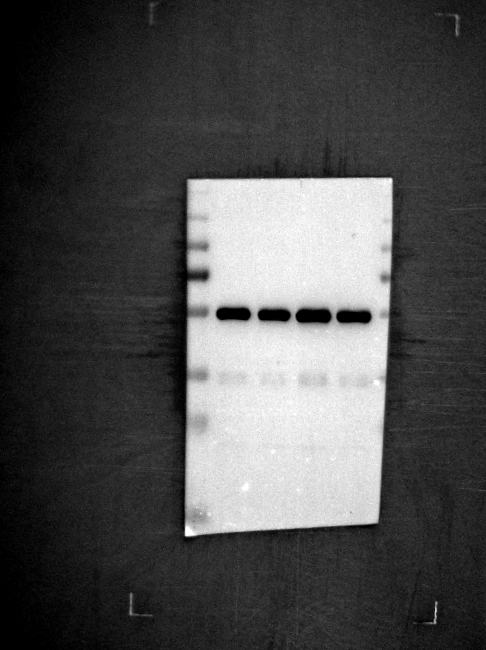

Supplement: Supplementary file 5 [file Image3.tif]

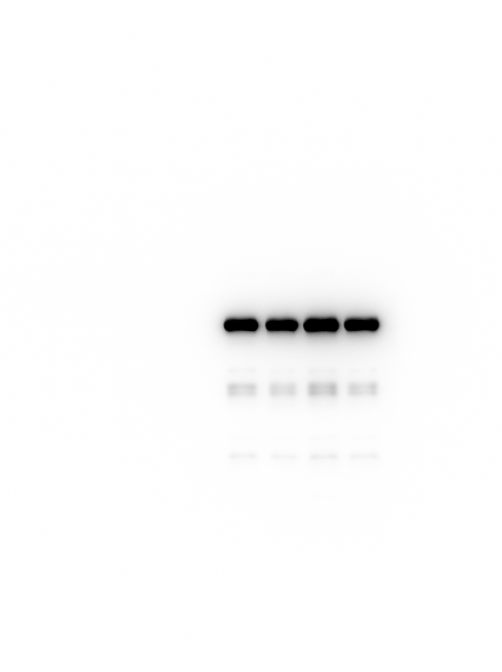

Supplement: Supplementary file 6 [file Image4.tif]

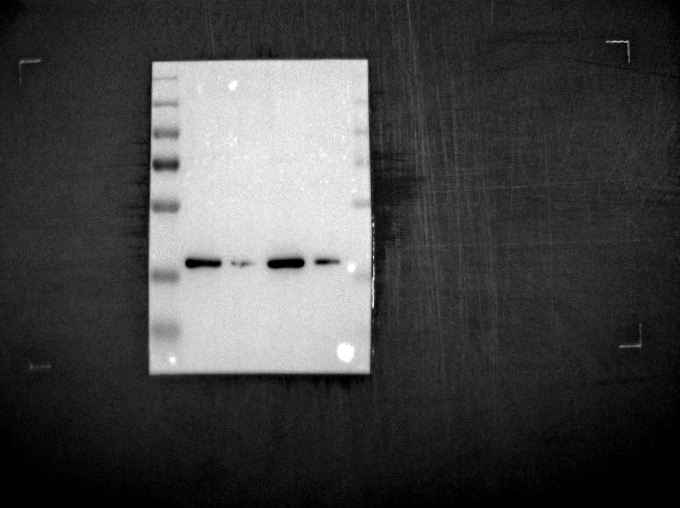

Supplement: Supplementary file 7 [file Image5.tif]

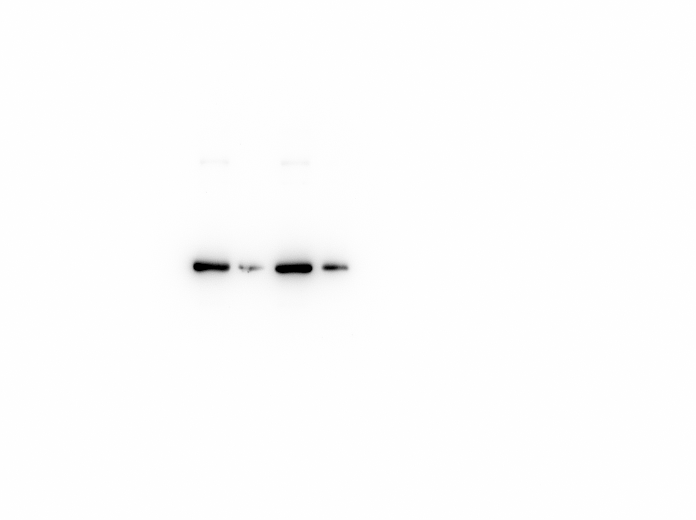

Supplement: Supplementary file 8 [file Image6.tif]

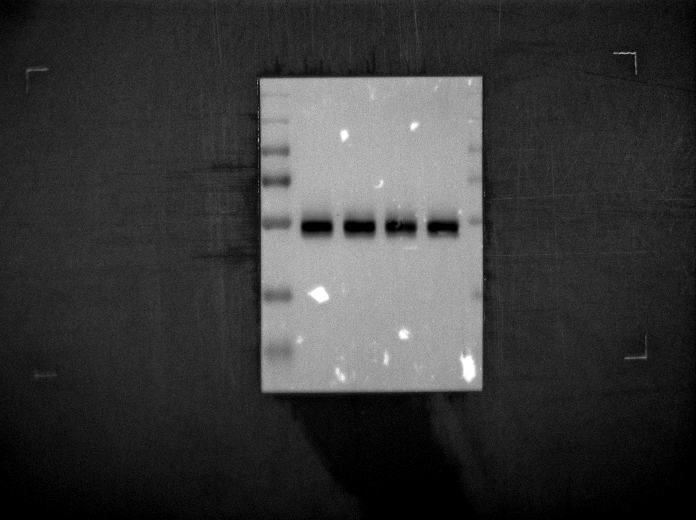

Supplement: Supplementary file 9 [file Image7.tif]

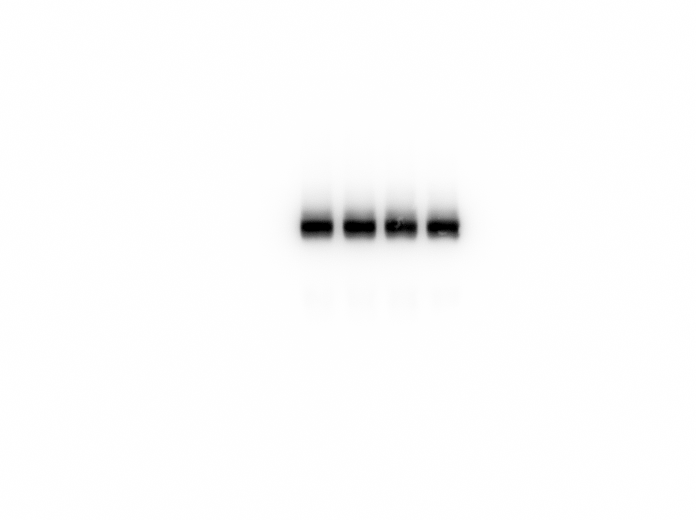

Supplement: Supplementary file 10 [file Image8.tif]

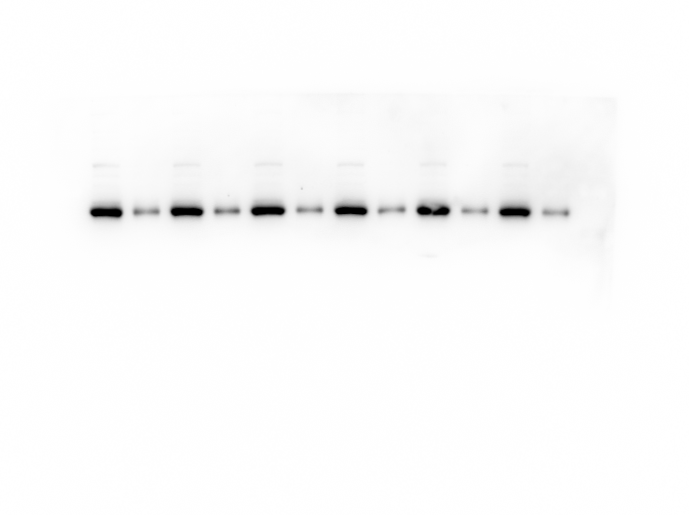

Supplement: Supplementary file 11 [file Image9.tif]

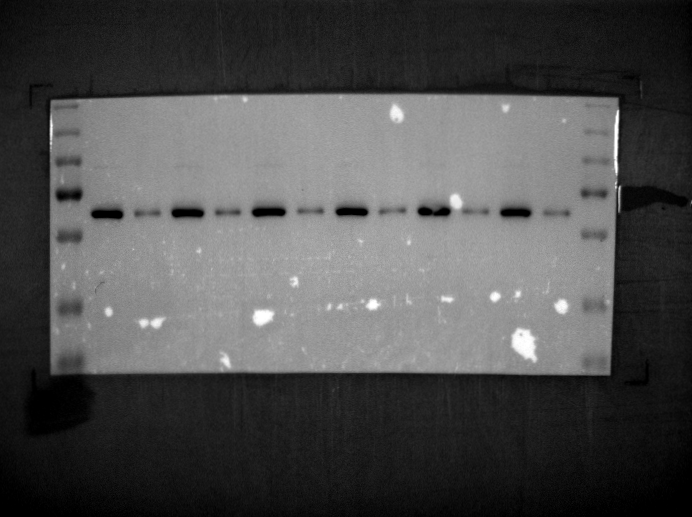

Supplement: Supplementary file 12 [file Image10.tif]

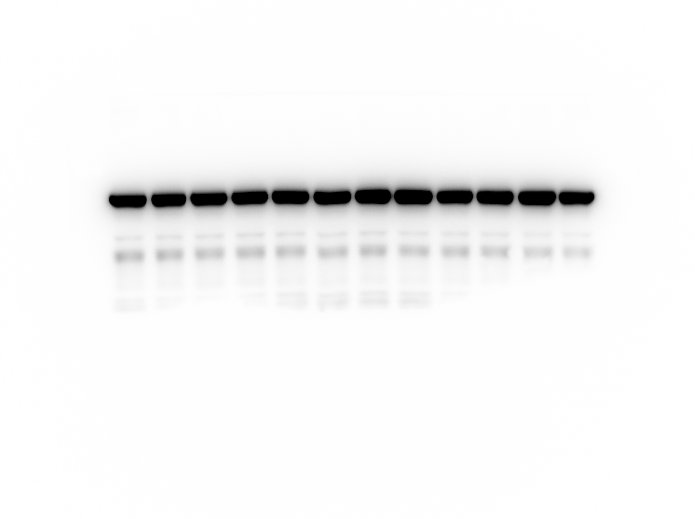

Supplement: Supplementary file 13 [file Image11.tif]

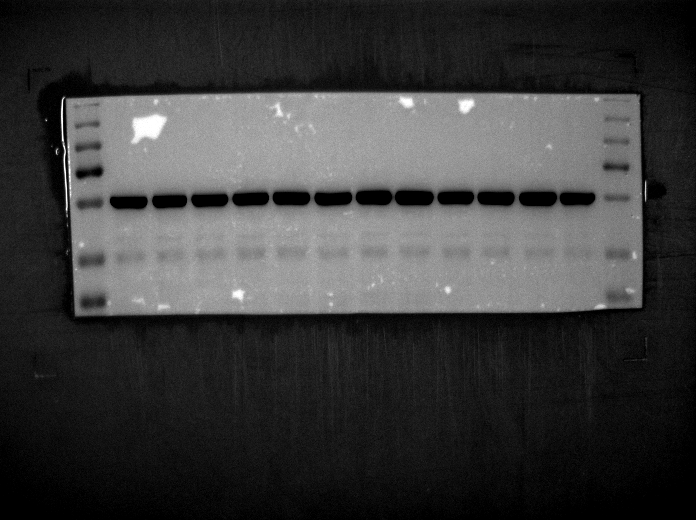

Supplement: Supplementary file 14 [file Image12.tif]

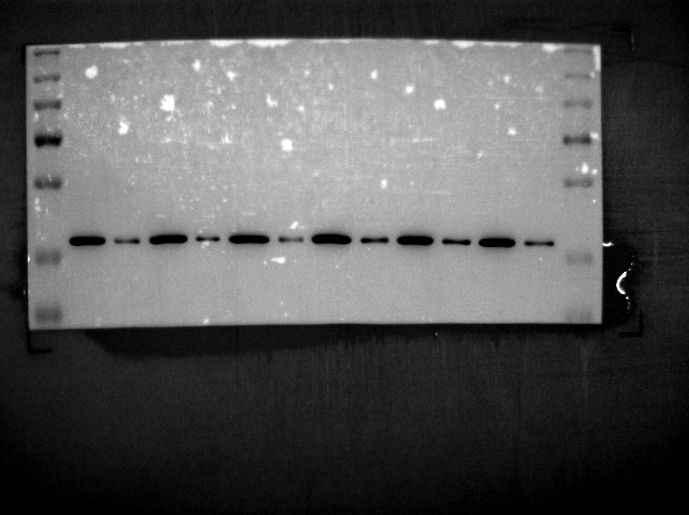

Supplement: Supplementary file 15 [file Image13.tif]

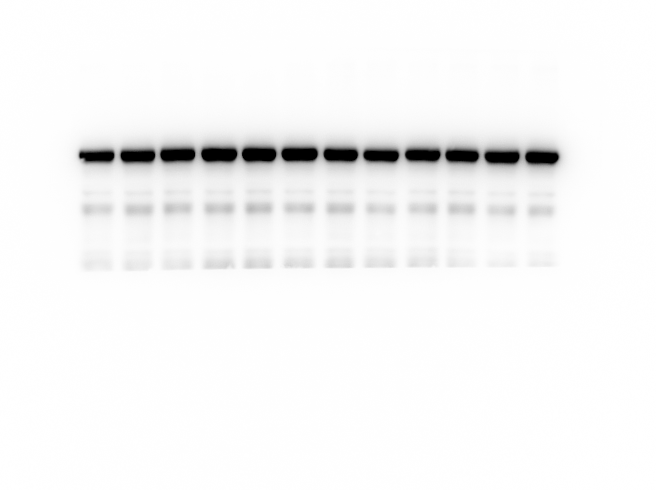

Supplement: Supplementary file 17 [file Image15.tif]
